# *Escherichia coli* is poised to grow using 5′-deoxynucleosides via MtnR and CRP regulation of DHAP shunt gene expression

**DOI:** 10.1128/jb.00280-25

**Published:** 2025-10-16

**Authors:** Katherine A. Huening, Caitlin C. Wingerd, Joshua T. Groves, Katelyn T. Kapusta, Laiba Khan, F. Robert Tabita, Justin A. North

**Affiliations:** 1 Department of Microbiology, The Ohio State University 215854, Columbus, Ohio, USA; Department of Microbiology and Cell Science, University of Florida, Gainesville, Florida, USA

**Keywords:** extraintestinal pathogenic *E. coli*, gene regulation, carbon metabolism, nucleosides, carbon catabolite repression

## Abstract

The dihydroxyacetone phosphate (DHAP) shunt is a multifunctional pathway for the metabolism of 5′-deoxynucleosides and 5-deoxypentose sugars, such as 5′-methylthioadenosine (MTA) and 5′-deoxyadenosine (5dAdo), into DHAP and an aldehyde species depending on the substrate. Previous work revealed that *Escherichia coli* strains with the DHAP shunt can utilize exogenous MTA, 5dAdo, and derivatives thereof as sole carbon and energy sources for growth. However, if and how the DHAP shunt was regulated for 5′-deoxynucleoside and 5-deoxypentose sugar metabolism remained unknown. In the present work, the DHAP shunt genes (*mtnK*, *mtnA*, and *ald2*) and a putative transporter gene of *E. coli* ATCC 25922 are observed to form an operon, which can be expressed from two separate transcription start sites (TSSs). The distal, low-activity TSS appears to be constitutive, while the proximal primary TSS is regulated based on the identity of available growth substrates by at least two transcriptional regulators. First, YjhU, a deoxyribonucleoside operon repressor family regulator previously of unknown function that we designate as MtnR, functions as a repressor of the DHAP shunt operon when DHAP shunt substrates are absent. Further, the cyclic AMP receptor protein imposes carbon catabolite repression while glucose is available. Based on comparative sequence analysis, the *E. coli* DHAP shunt promoter region is highly conserved, including strains of the globally disseminated ST131 lineage of extraintestinal pathogenic *E. coli*, indicating a similar regulatory paradigm. Thus, the *E. coli* DHAP shunt is a previously unrecognized pathway for the use of 5′-deoxynucleosides and 5-deoxypentose sugars as alternative carbon sources when glucose is scarce.

**IMPORTANCE:**

While not found in all *Escherichia coli* strains, the dihydroxyacetone phosphate (DHAP) shunt pathway is present in multiple lineages of extraintestinal pathogenic *E. coli*. The DHAP shunt allows *E. coli* strains to use a range of 5′-deoxynucleosides and 5-deoxypentose sugars as carbon and energy sources. These metabolites were previously considered waste products of cellular metabolism. This study identifies two transcriptional regulators that regulate the DHAP shunt operon, only allowing full expression when a DHAP shunt substrate is present and when glucose, a more-preferred carbon substrate, is absent. This demonstrates that the DHAP shunt is a genuine carbon metabolism pathway in *E. coli* and is placed under the hierarchy of carbon catabolite repression.

## INTRODUCTION

Regardless of the environment, bacteria must acquire nutrients and generate energy to grow. As such, many microbes are metabolically diverse to adapt and survive in dynamic or varied environmental settings (1–3). In *Escherichia coli*, expression of numerous genes involved in the metabolism of preferred and alternative growth substrates is modulated by multiple regulatory mechanisms. For example, when glucose is limiting, expression of the *ptsGHI* and *malXYZ* phosphotransferase systems (PTS) for glucose and maltose transport and phosphorylation is tuned through action of the Mlc repressor protein and cyclic AMP receptor protein (CRP) (4). This prevents wasteful overproduction of the PTS transporters for glucose while still positioning the cell to resume glucose uptake when available in the future (4). PTS systems operate by first transferring the phosphoryl group from phosphoenolpyruvate to enzyme I (ptsI) and then HPr (histidine protein; *ptsH*), which function independently of the growth substrate(s) available (5). The phosphate is then transferred to one of at least 23 different substrate-specific EII PTS systems (4). For example, EIIBC^Glc^ (*ptsG*) transports glucose into the cell, and then phosphorylated EIIA^Glc^ (catabolite repression resistance protein, also known as EIII^Glc^; *crr*) transfers its phosphate to glucose forming glucose-6-phosphate (5, 6). Also, EIIA^Glc^ regulates the synthesis of cyclic AMP (cAMP) for CRP activation. Limiting glucose results in a buildup of phosphorylated EIIA^Glc^, which in turn stimulates synthesis of cAMP by adenylate cyclase (*cyaA*). The EIIA^Glc^ phosphorylation state and resulting cAMP levels regulate CRP activity for expression of alternative growth substrate metabolism genes—a process termed carbon catabolite repression (CCR). Over 100 different promoters for genes are regulated by CRP, including those involved in the metabolism of alternative growth substrates, such as fucose, lactose, arabinose, and glycerol (6, 7). EIIA^Glc^ can also perform inducer exclusion by directly interacting with and inhibiting the activity of metabolic enzymes and transporters of alternative growth substrates (6). In addition to CCR and inducer exclusion mechanisms, expression of genes for metabolism of alternative growth substrates can be regulated through specific transcriptional regulators that are often allosterically controlled, such as LacI repressor for the well-characterized Lac operon (6, 8). The combination of these mechanisms enables *E. coli* to prioritize the use of preferred carbon sources like glucose over alternative growth substrates. As an exception, the Entner-Doudoroff pathway for conversion of gluconate to pyruvate for carbon and energy metabolism is continuously expressed in *E. coli* (9). Ultimately, it is advantageous for versatile microbes such as *E. coli* that inhabit a myriad of dynamic environmental niches, including the gut, blood, and bladder, to be able to regulate gene expression for the catabolism of alternate growth substrates in response to nutrient fluctuations, particularly when preferred substrates like glucose and gluconate become limiting (10).

**Fig 1:**
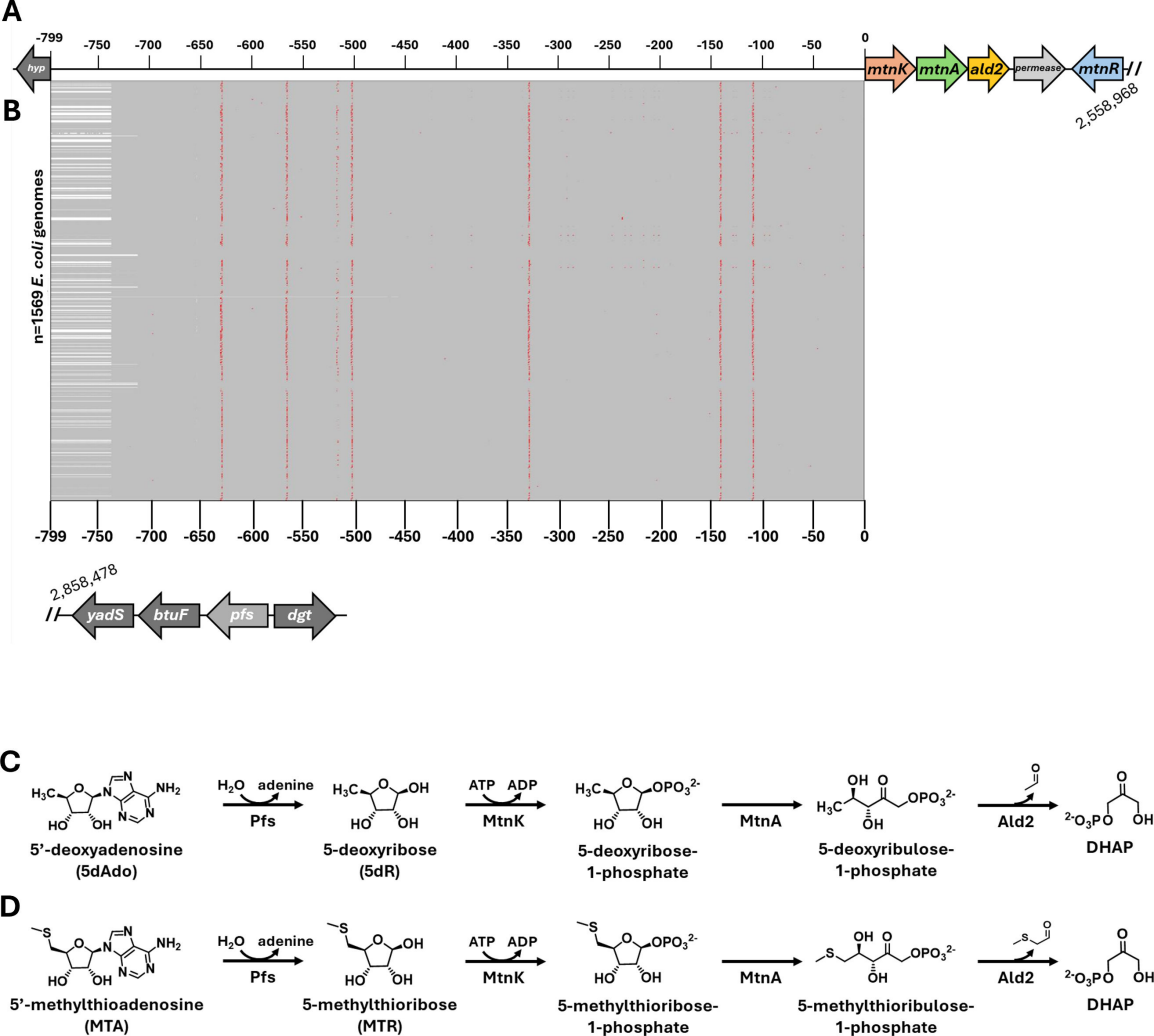
The multi-functional DHAP shunt of *E. coli*. (**A**) Genomic organization of the DHAP shunt operon and neighboring genes in *E. coli* ATCC 25922. The operon specifically consists of *mtnK*, *mtnA*, *ald2*, and an annotated permease of unknown function (Fig. S1B). The *pfs* gene, conserved across all *E. coli*, is encoded in a different region of the *E. coli* genome. (**B**) Multiple sequence alignment of the DHAP shunt 5′-UTR region from 1,569 *E. coli* genomes containing the DHAP shunt operon. Positions matching the ATCC 25922 reference are shown in gray, while mismatches are shown in red. (**C and D**) Biochemical reactions catalyzed by the DHAP shunt pathway enzymes, with 5′-deoxyadenosine (**C**) or 5′-methylthioadenosine (**D**) as initial substrate.

A recently discovered metabolic pathway, the dihydroxyacetone phosphate (DHAP) shunt, has been shown to enable *E. coli* strains that possess it to grow using 5′-deoxynucleosides and 5-deoxypentose sugars as sole carbon and energy sources (Fig. 1C and D) (11, 12). This includes 5′-methylthioadenosine (MTA) and 5′-deoxyadenosine (5dAdo) that arise as ubiquitous byproducts of *S*-adenosyl-l-methionine utilization, and 5-methylthioribose (MTR) and 5-deoxyribose (5dR) that are subsequently produced by MTA/5dAdo nucleosidases (MtnN; Pfs in *E. coli*) (13). The *E. coli* variation of the DHAP shunt, as characterized in the attenuated extraintestinal pathogenic *E. coli* (ExPEC) strain ATCC 25922 (Seattle 1949) (14–16), is composed of 5-methylthioribose/5-deoxyribose kinase (*mtnK*), 5-methylthioribose-1-phosphate/5-deoxyribose-1-phosphate isomerase (*mtnA*), and 5-methylthioribulose-1-phosphate/5-deoxyribulose-1-phosphate aldolase (*ald2*, a class II aldolase). In *E. coli*, the DHAP shunt genes and a putative permease gene are clustered together on the tRNA-leuX genomic island (Fig. 1A, C and D) (12). Directly upstream of the DHAP shunt genes in ATCC 25922 is an intergenic region of 799 bp in length, with no other known open reading frames, and it is heavily conserved across *E. coli* containing the DHAP shunt gene cluster (Fig. 1B; Fig. S1A). Among *E. coli*, the DHAP shunt is primarily observed in ExPEC, including the globally disseminated ST131 lineage. ExPEC strains are responsible for infections in the bloodstream, cerebral fluid, and urinary tract (17). In contrast, <0.1% of enteropathogenic *E. coli* appear to have the DHAP shunt (12). Notably, the physiological role of the DHAP shunt varies. In organisms such as *Rhodospirillum rubrum* and *Rhodopseudomonas palustris*, the DHAP shunt facilitates the use of MTA and MTR as sulfur sources via the resulting 2-methylthioacetaldehyde, and in *Bacillus thuringiensis*, the DHAP shunt prevents inhibitory 5dR accumulation. Neither of these roles has been observed in *E. coli* (11, 12, 18–20). In *R. rubrum*, the DHAP shunt genes are constitutively expressed at low levels, consistent with a housekeeping function in sulfur salvage from MTA (12, 20).

Outside of the ubiquitous *E. coli pfs* gene, regulation of the DHAP shunt in *E. coli* has remained entirely unknown (13, 21). Here, we demonstrate that ExPEC possessing the DHAP shunt controls its expression through CCR to activate expression when glucose is limiting, and a second transcriptional regulator, YjhU, that we designate as MtnR, for DHAP shunt repression when DHAP shunt substrates are absent. This dual regulation allows *E. coli* to express the DHAP shunt only after glucose becomes limiting and DHAP shunt substrates are present. Moreover, this establishes that in ExPEC, the DHAP shunt is part of the hierarchy of alternative carbon metabolism pathways that are reserved for use after preferred substrates like glucose and gluconate are depleted. Given extraintestinal environments like the urinary tract are typically limited in glucose availability (<250 µM) (22, 23) and rather are composed of modified amino acids, nucleosides, and sugars, it is expected that ExPEC can turn to MTA and 5dAdo as growth substrates to aid survival and growth in glucose-poor environments (1, 22, 24–26).

## MATERIALS AND METHODS

### Chemicals

5-deoxy-d-ribose (5dR) was supplied from either Millipore-Sigma or Santa Cruz Biotechnology, Inc. Concentrations (5–8 mM) used in growth experiments were dependent on the supplier and were selected to yield similar growth results. All other chemicals were purchased from Millipore-Sigma, unless otherwise stated.

### Bacterial strains and general growth

*E. coli* clinical isolate ATCC 25922, which natively contains the DHAP shunt genes, was the primary strain used in this study (27). Deletion of *crp* and *mtnR* (previously *yjhU*) in *E. coli* ATCC 25922 was performed using the λ-red recombinase system as previously described (11, 28). Primers used for PCR amplification from pKD4 for gene deletion are listed in Table S2. *E. coli* Top10 (Invitrogen) and Stellar (Takara Biosciences) were used for cloning and plasmid storage. All transformations of ATCC 25922 strains were performed by electroporation. For routine culturing, *E. coli* was grown in either lysogeny broth or modified M9 media with carbon source as indicated and as previously described (11) with appropriate antibiotics: 100 µg/mL streptomycin for strains with pLacZ and pTETTET series of plasmids and 50 µg/mL kanamycin for *mtnR* and *crp* deletion strains. Sodium nitrate was omitted unless otherwise indicated. Cultures were grown aerobically at 37°C with shaking at 250 rpm.

### Plasmid construction

Plasmids for *lac*Z expression under control of the DHAP shunt promoter fragments were constructed from pBBRsm2-MCS5 (29), where the *lacZα* gene within the multiple cloning site was replaced by the *E. coli lacZ* gene fused to various fragments of the DHAP shunt promoter region or the *lac* promoter, as fully detailed in the Supplemental methods. The *E. coli* complementation vector pTETTET was used to express *crp* or *mtnR* from a tetracycline inducible promoter in the ATCC 25922 Δ*crp* or Δ*mtnR* strains, respectively, as previously described (11). The CRP complementation plasmid, pCRP, was constructed using CRP-NdeI_F and CRP-AgeI_R primers to amplify the *crp* gene while introducing flanking NdeI and AgeI restriction sites for cloning into the pTETTET vector (Table S1). Similarly, pMtnR was constructed by introducing NdeI and SacI restriction sites by PCR that flanked the *mtnR* gene using primers mtnR-NdeI_F and mtnR-SacI_R (Table S1) and cloning into the pTETTET vector. For CPR production, plasmid pET28-crp was constructed using primers CRP-NdeI_F and CRP-HindIII_R to amplify the *crp* gene and introduce flanking NdeI and HindIII restriction sites, which were used to clone into the NdeI and HindIII restriction sites of pET28a (Invitrogen, Table S1).

### Growth studies

*E. coli* strain ATCC 25922, Δ*mtnR*, Δ*mtnR* + pMtnR, Δ*crp*, and Δ*crp* + pCRP were grown aerobically at 37°C in M9 media with 5 mM glucose to mid-exponential phase and washed three times in M9 without a carbon source. The washed pellet was resuspended in carbon-free M9, diluted 1:10, and used to inoculate culture tubes or wells of a 96-well plate (Falcon) containing 200 µL of M9 with either 1 or 5 mM glucose, 5–8 mM 5dR, 0.09% wt/vol glycerol, 25 mM lactose, 5 mM fucose, 5 mM rhamnose, or 5 mM N-acetylneuraminic acid (NANA). Tetracycline at 50 ng/mL was included for induction of *crp* expression from pCRP, and 10 ng/mL tetracycline was included for induction of *mtnR* expression from pMtnR (Fig. S4A). Plates were incubated at 37°C for 24 h in a SpectraMax iD3 plate reader, and OD_600_ and OD_420_ measurements were recorded every 30 min. Aerobic growth was maintained by setting the plate to shake for each 30 min interval and briefly pausing while collecting the optical density (OD) readings at the indicated wavelenngth in nm. Final OD readings were averaged for *n* = 5 independent replicates, unless otherwise noted. Tubes were incubated at 37°C with 250 rpm shaking until the OD_600_ reached the middle of the exponential growth phase for further enzyme activity and transcript analysis studies. Mid-exponential growth was observed at OD_600_ = ~0.2–0.3 for cultures with 5–8 mM 5dR as carbon source, ~0.4–0.5 for cultures containing 5 mM fucose or rhamnose as carbon source, or ~0.5–0.6 for cultures containing 5 mM arabinose, NANA, or glucose.

### LacZ activity assays

Cultures of ATCC 25922 or ATCC 25922 Δ*mtnR* containing either placZ, placZ799, placZ255, placZ169, placZ126, placZ66, placZUR1, placZUR2, or placZUR3 (Table S1) were grown in M9 minimal medium with 40 mM sodium nitrate (does not affect aerobic growth [11]) and indicated carbon source to mid-exponential phase. For LacZ activities under glucose starvation, *E. coli* ATCC 25922 containing placZ255 was grown in M9 minimal media with 5 mM glucose as the sole carbon source. Once cultures reached mid-exponential phase, all cultures were washed in carbon-free M9 medium. Half of the cultures were resuspended in M9 medium with 5 mM glucose, while the other half were resuspended in M9 lacking a carbon source, then further incubated at 37°C, 250 rpm, for an additional 40 min before harvesting. Cells were harvested by centrifugation at 5,000 × *g* for 5 min, and cell extracts were prepared by sonication and assayed as described previously (30, 31) with modifications fully detailed in the Supplemental methods. Statistical analysis was performed using the two-tailed *t*-test.

### RNA extraction

Cultures of *E. coli* ATCC 25922 strains grown for RNA extraction were aerobically incubated at 37°C in M9 media supplemented with the specified carbon source to mid-exponential phase. Cultures were stored in RNAprotect (Qiagen) until cells could be lysed using chemical (Tris-EDTA 15 mg/mL lysozyme solution) and physical (bead beating) methods. RNA was extracted from cell lysate using the Qiagen RNeasy kit (Qiagen).

### Transcription start site mapping

Regions upstream of the DHAP shunt operon were amplified from *E. coli* ATCC 25922 genomic DNA as templates for sequencing ladders. These regions included fragments spanning 381–709 bp upstream of *mtnK* (fragment 1; primers Frag1_F 2.1 and Frag1_R 2.1), 138–433 bp upstream of *mtnK* (fragment 2; primers Frag2_F 2.0 and Frag2_R 2.0), and 104 bp upstream to 45 bp within the *mtnK* coding sequence (fragment 3; primers USP-92-AseF and MtnK-rev 2.0) (Table S2; Fig. S5A). PCR amplification with dideoxynucleotide (ddNTP) termination was conducted using ddNTPs (AAT Bioquest dideoxynucleotides; ddATP, ddTTP, ddGTP, and ddCTP) added individually to separate reactions at a ratio of 1:20 (dNTP:ddNTP). Amplification of the sequencing ladders and transcription start site (TSS) mapping via primer extension were performed using Vent (exo-) polymerase following the manufacturer’s protocol (New England Biolabs) with the 5′-Cy5-labeled primers mtnK-rev-Cy5 (fragment 3), Frag2_R 2.0 CY5 (fragment 2), and Frag1_R 2.1 CY5 (fragment 1) (Table S2). Template DNA used for primer extension was prepared from RNA extracted from cells grown as described under Growth Studies and reverse transcribed using Quantabio qScript Ultra SuperMix to create a complementary DNA (cDNA) pool. All sequencing ladders and primer extension reactions for TSS mapping were resolved by 15% or 6% acrylamide/urea denaturing gel and imaged on a Typhoon Imager (GE Amersham, Cytive) using the Cy5 fluorescent filter.

### Transcriptomic analysis

Cultures of *E. coli* ATCC 25922 and ATCC 25922 Δ*mtnR* were grown as described under Growth Studies in M9 medium with either 5 mM glucose or 5 mM 5dR as the sole carbon source. Cultures were harvested at mid-exponential phase, and RNA was extracted as described above. For carbon starvation transcriptomic analysis, cultures of *E. coli* ATCC 25922 were grown aerobically at 37°C, 250 rpm in M9 10 mM glucose to mid-exponential phase and washed three times with M9 without carbon. The resulting cultures were resuspended in either M9 without carbon (*n* = 2), to simulate carbon starvation conditions, or M9 with 10 mM glucose (*n* = 2). After a 40 min aerobic incubation at 37°C, cells were harvested, and RNA was extracted.

To analyze the global transcript pool, cDNA libraries were prepared from extracted RNA using the Illumina Stranded Total RNA Prep, Ligation with Ribo-Zero Plus kit (Illumina). Libraries were sequenced using a NextSeq 2000 sequencer (Illumina). Trimming was performed using Trimmomatic (32). Alignment of sequences to the reference genome was performed using Bowtie2 (33). Differential comparison was made using DESeq2 (34). Transcriptomic reads were mapped to ATCC 25922 reference sequences NZ_CP032085.1 (chromosome), NZ_CP032087.1, NZ_CP032088.1, and NZ_CP032086.1 (plasmids). Transcriptomic data were categorized based on annotations and functional descriptions from the UniProt and NCBI databases. Genes that were upregulated >2.5 log_2_-fold or downregulated <−2.5 log_2_ were included in the comparison. Unannotated genes or genes with unknown function were not included in the carbon starvation analysis.

### qRT-PCR

Extracted RNA was treated with DNase I (Invitrogen) following the manufacturer’s instructions. DNA-free RNA was used to generate a cDNA library using qScript Ultra SuperMix (Quantabio) following the manufacturer’s instructions. The quantative Real Time - Polymerase Chain Reaction (qRT-PCR) reactions were performed with a 7500 Fast Real-Time PCR System (Applied Biosystems, Thermo Fisher Scientific) using iQ SYBR Green Supermix (BioRad) following the manufacturer’s instructions with 125 nM of each primer and 0.2 ng/μL cDNA template were used. The gene of interest for qRT-PCR was either *lacZ* (using primers qRT_lacZ_F and qRT_lacZ_R; Table S2) or *mtnK* (using primers qRT_mtnK_F and qRT_mtnK_R), with a reference gene of *rpoD* (using primers qRT_rpoD_F and qRT_rpoD_R). Primer pairs were analyzed for amplification efficiency (35), and all primer pairs had a percent efficiency above 78%. Fold change in gene transcript between treatment and control conditions relative to *rpoD* was calculated using the ΔΔCt method (36, 37). Statistical analysis was performed using the two-tailed *t*-test.

### Comparative genomic analysis

To identify upstream UTRs with sequence similarity to the 799 bp region upstream of the DHAP shunt operon in *E. coli* strain ATCC 25922 (Table S3), the sequence was queried against *E. coli* genomes using NCBI BLASTn. Parameters were adjusted to return up to 5,000 hits and to reduce penalties for mismatches and gaps. Python scripts were used to align and evaluate the reference coverage of each sequence (Supplemental methods). Genomic sequences corresponding to these hits were retrieved using NCBI’s Batch Entrez and assessed for a complete DHAP shunt operon (Dataset S3). For genomes containing at least 75% of the operon, the identified upstream regions were aligned to the 799 bp reference sequence from ATCC 25922 to perform a position-specific percent identity analysis (Dataset S2).

### CPR purification and electrophoretic mobility shift assays

Hexahistidine-tagged *E. coli* CRP protein was produced in *E. coli* BL21 (DE3) carrying the expression plasmid pET28-crp, similar to procedures previously described (38) and fully detailed in the Supplemental methods. Binding of CRP to target DNA sequences was performed by electrophoretic mobility shift assay (EMSA) using 5% 29:1 linear:bis-acrylamide gel with 0.3× Tris-Borate-EDTA buffer and 150 V electric field at 20°C. Cy5-labeled and unlabeled P1/2 promoter region containing promoters P1 with the anticipated CRP binding site and P2 was amplified using primers Footprint_F and Footprint_R or Footprint_R-Cy5 (Table S2). Unlabeled DNA fragments of the *rpoD* gene and the region upstream of P1 and P2 (UR2, Fig. 3A) were amplified by PCR using primers qRT_rpoD_F and qRT_rpoD_R, and UR2-799_F and UR1-SpeI_R, respectively (Table S2). Each EMSA reaction (20 μL) contained 20 mM Tris, 10 mM MgCl_2_, 0.1 mM EDTA, 100 mM KCl, 200 μM cAMP, 0–300 nM CRP, and DNA. Each reaction contained either 22 nM of a 10:1 ratio of unlabeled:Cy5-labeled P1/2 DNA for CRP binding assays, or 202 nM of a 100:1 ratio of unlabeled:Cy5-labeled DNA for competition assays as described in Fig. 7 legend. The reactions were incubated at room temperature for 15 min prior to analysis by EMSA. Gel images were acquired by Typhoon Imager using the Cy5 filter.

**Fig 2:**
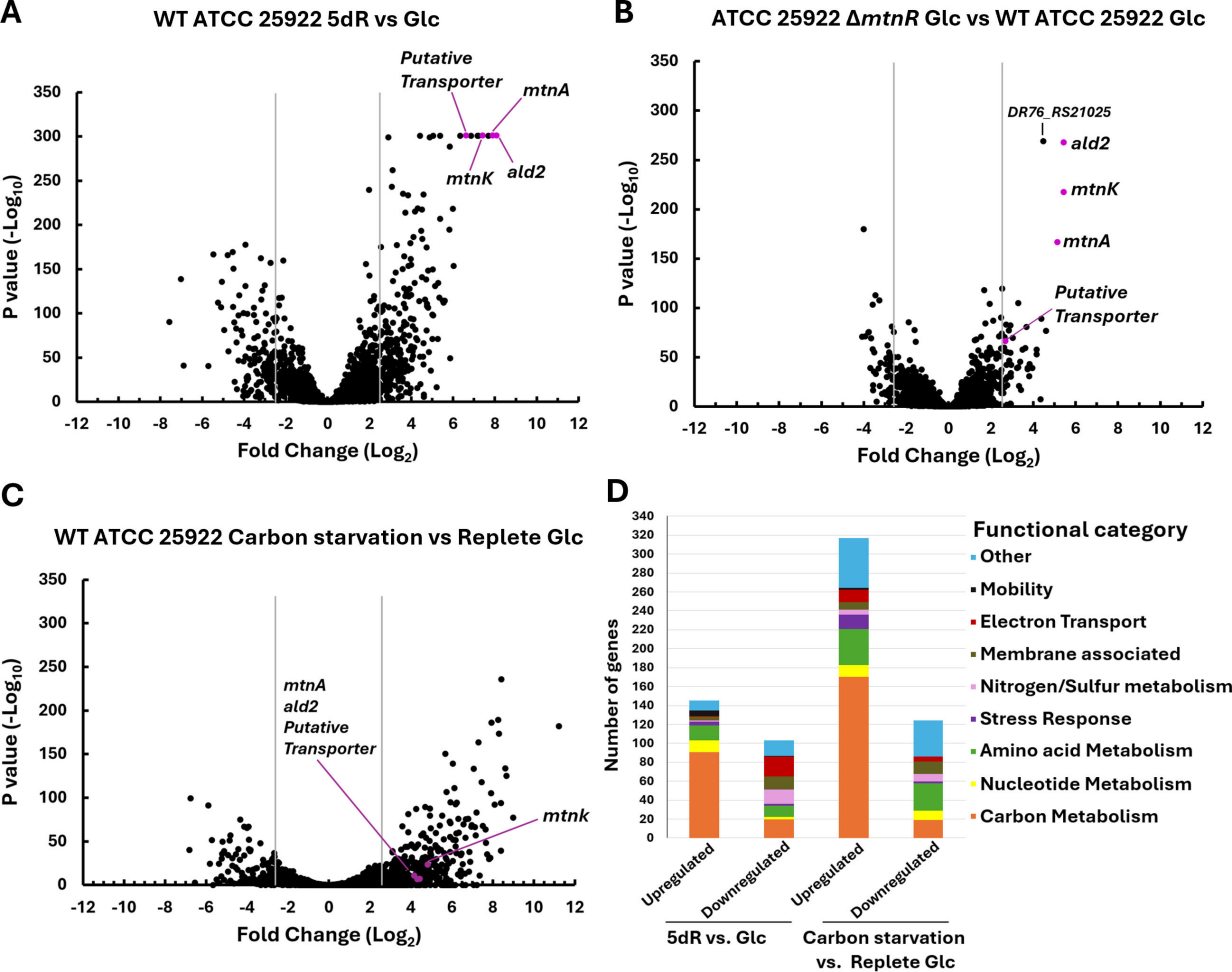
Regulation of *E. coli* ATCC 25922 transcriptome during growth on glucose, 5dR, or carbon starvation. Volcano plots of changes in ATCC 25922 transcript abundance for (**A**) wild-type ATCC 25922 grown with 5dR versus glucose, (**B**) MtnR deletion strain (∆*mtnR*) versus wild-type ATCC 25922 grown with glucose, and (**C**) ATCC 25922 incubated under carbon starvation conditions versus carbon replete (glucose) conditions. Gray lines indicate 2.5 log_2_-fold change thresholds; DHAP shunt genes are highlighted in pink. (**A**) DHAP shunt genes are strongly upregulated (~7.5 log_2_) in the presence of 5dR. (**B**) Deletion of MtnR (∆*mtnR*) results in ~5 log_2_ increased expression of DHAP shunt genes compared to the wild-type strain in the presence of glucose. (**C**) Carbon starvation only moderately upregulated DHAP shunt gene expression. (**D**) Inventory of genes differentially expressed under 5dR growth and carbon starvation relative to growth on glucose. Carbon starvation triggers a large change in the proteome compared to growth on 5dR, indicating the proteome change during growth on 5dR is specific to the substrate and not a general stress response.

## RESULTS

### Transcriptomic analysis reveals that DHAP shunt genes are regulated in response to 5dR versus glucose availability

To determine if the DHAP shunt and other genes in *E. coli* strain ATCC 25922 were regulated for growth on DHAP shunt substrates versus glucose, transcriptomic analysis was performed comparing WT ATCC 25922 grown with 5dR or glucose as sole carbon sources. This revealed significant changes in the expression of multiple genes (Fig. 2A). A total of 392 out of 4,732 genes were differentially regulated between conditions (≥2.5 log_2_-fold change), with 122 genes downregulated and 257 genes upregulated in cultures grown on 5dR versus glucose. Of the upregulated genes, more than half of the annotated genes are associated with cellular carbon metabolism, particularly of alternative growth substrates (Fig. 2D). This included N-acetylneuraminic acid metabolism and transport (*nanATEK*, *nanS*, *nanC*), glycerol metabolism and transport (*glpFK*, *glpQ*, *glpT*), gulonic acid transport (*yiaM*, *yiaN*, *yiaO*, *yiaK*), and acetate metabolism (*fadABDEHIJK*, *aceBA*, *glcABCDEF*) (Fig. S2A; Dataset S1). Notably, the genes with the most change in abundance (~7.5 log_2_-fold) were the DHAP shunt genes: *mtnK*, *mtnA*, *ald2*, and a putative permease gene located immediately next to *ald2* (Fig. 1A). Analysis of the read depth from the transcriptomics RNA sequencing revealed that each gene associated with the DHAP shunt gene cluster (*mtnK*, *mtnA*, *ald2*, and the putative transporter) exhibits a similar number of reads. The similar read depth and expression fold change for each gene suggested that the four DHAP shunt genes are transcribed as an operon (Fig. S1B) (39).

Given the upregulation of several alternative carbon metabolic pathways in the transcriptomic analysis, in addition to the DHAP shunt, we sought to determine whether the strong induction of the DHAP shunt during growth on 5dR versus glucose was specific to 5dR or a general carbon starvation response. In *Bacillus subtilis*, genes for the universal methionine salvage pathway metabolize MTR back to methionine primarily under carbon starvation conditions, but also to a lesser extent under sulfur and nitrogen starvation (40). To differentiate between DHAP shunt regulation in the presence of 5dR versus general carbon starvation, a second transcriptomic analysis compared WT ATCC 25922 that was initially grown on glucose and then shifted to conditions with or without glucose to initiate carbon starvation responses (Fig. 2C), similar to previous studies with *E. coli* K12 (41). The shift in *E. coli* ATCC 25922 from replete glucose to carbon starvation revealed more changes to the cell transcriptome than growth on 5dR versus glucose, with a total of 867 genes differentially expressed (≥2.5 log_2_-fold change). Of those, 171 genes were downregulated, and 696 genes were upregulated during carbon starvation versus replete glucose. Like growth on 5dR, about half of the upregulated annotated genes were associated with carbon metabolism, including metabolic pathways for alternative growth substrates and fatty acid degradation (Fig. 2D). Compared to glucose, expression of the DHAP shunt only increased ~4.5 log_2_-fold during carbon starvation, which is ~3 log_2_-fold less than growth on 5dR versus glucose (~7.5 log_2_-fold increase). During carbon starvation, but not 5dR growth, genes of the Lac operon (*lacZ*, *lacY*; lactose), Rha operon (*rhaSR*; rhamnose), ascorbate import and metabolism (*ulaABC* and *ulaDE*), fructose transport (*frwBCD*), and ethanolamine degradation (*eutT*, *eutP*, *eutQ*) were observed to be upregulated (Fig. S2B; Dataset S1). Furthermore, genes involved in acetate metabolism (β-oxidation of fatty acids, glyoxylate metabolism, and glyoxylate bypass) exhibited the highest upregulation during carbon starvation, but only a modest upregulation during 5dR growth (e.g., 11 log_2_ versus 7 for *fadB*), consistent with previous reports (42). Also, several annotated stress response proteins that increased >3 log_2_-fold during carbon starvation were not observed to be upregulated during growth on 5dR. These included *bolA* (general stress response transcriptional regulator), *cspD* (cold shock protein), *iprA* (oxidative stress resistance), *asr* (acid shock protein), *rclR* (reactive chlorine stress response transcriptional regulator), and *uspBCDEF* (universal stress proteins), similar to previous reports (41). Altogether, this indicates that the DHAP shunt is regulated as a metabolic pathway specific for utilization of 5dR as a substrate and not as part of a general carbon starvation mechanism.

### The DHAP shunt operon 5′-UTR contains two promoters: a proximal regulated primary and a distal low-activity secondary promoter

Previous comparative genomic analyses revealed that *E. coli* with the DHAP shunt invariably possessed *mtnK*, *mtnA*, *ald2*, and a putative transporter gene located at the end of the tRNA-*leuX* genomic island, indicating a shared evolutionary origin of the operon among *E. coli* strains (12). Building on this, we performed sequence alignment of the 5′-UTR region of the DHAP shunt operon using the 799 bp intergenic region of *E. coli* ATCC 25922 between *mtnK* and the closest upstream ORF (DR76_R530330; RefSeq WP_000788354) as the reference (Fig. 1A). The vast majority of strains with the DHAP shunt exhibited the same ~799 bp 5′-UTR as ATCC 25922 (1,569 of 1,847 selected genomes) save for a few mismatches (Fig. 1B). Overall, we identified 52 variations in sequence coverage between 1,847 *E. coli* genomes and the 799 bp 5′-UTR reference sequence of ATCC 25922 (Fig. S1A). Strains with no alignment to the beginning of ATCC 25922 5′-UTR (Fig. S1A) exhibited truncation or loss of the DHAP shunt operon, often due to insertional sequences. Conversely, strains possessing the DHAP shunt operon all shared a minimal ~250 bp region from the beginning of the 5′-UTR, suggesting that key regulatory elements, including potential promoter sequences, are located within this segment.

**Fig 3:**
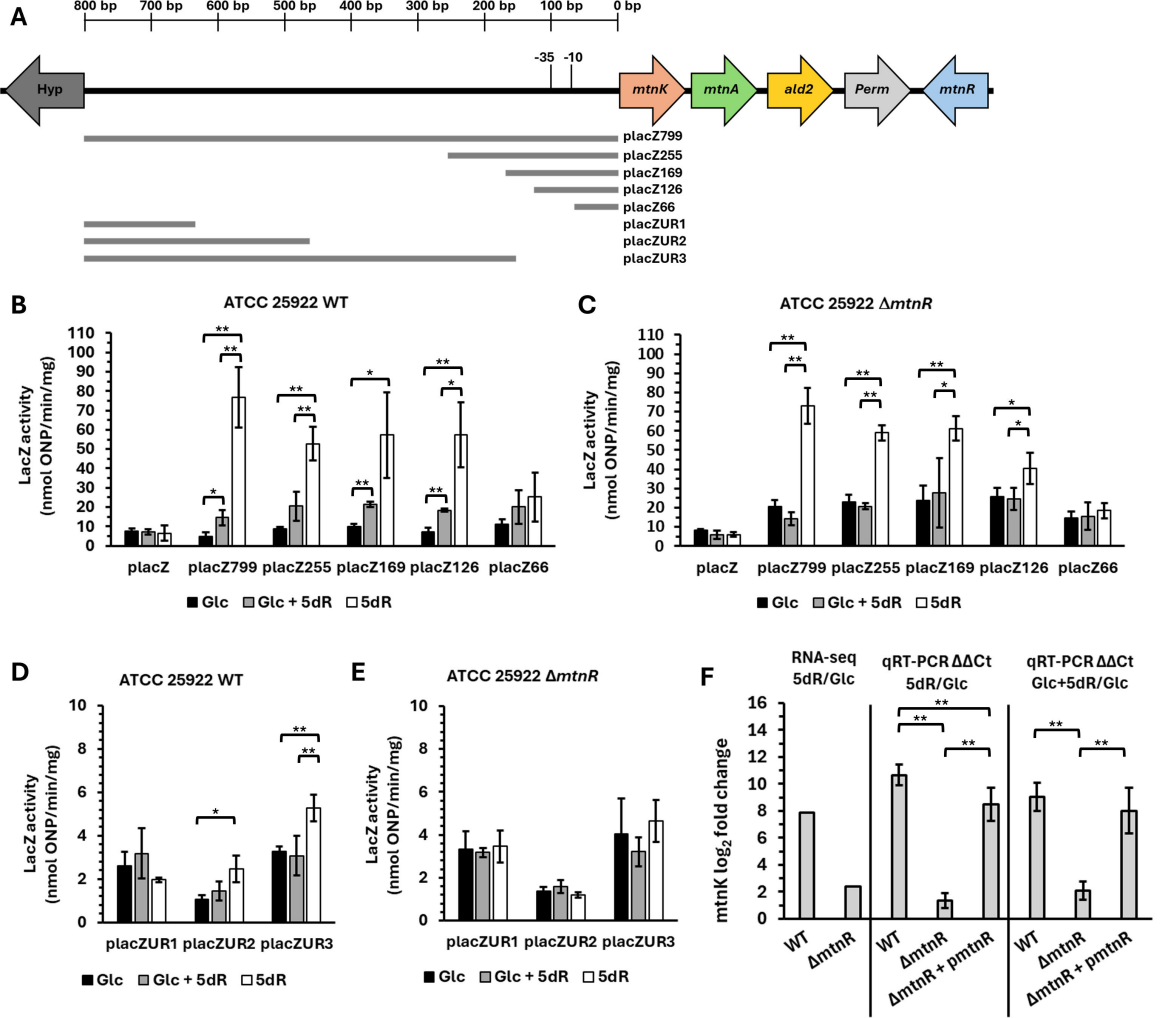
DHAP shunt 5′-UTR regions required for operon expression and repression by MtnR. (**A**) *E. coli* ATCC 25922 DHAP shunt genomic context and sections of 5′-UTR used for construction of plasmids (pLacZ###) with promoter–*lacZ* fusions. (**B, D**) LacZ activity assays from cell extracts of wild-type *E. coli* ATCC 25922 and (**C, E**) LacZ activity assays from cell extracts of MtnR deletion strain (Δ*mtnR*) containing the indicated DHAP shunt 5′-UTR–*lacZ* fusion plasmids when grown with either 5 mM glucose (Glc), 5 mM glucose plus 5 mM 5dR (Glc + 5dR), or 5dR as the carbon source. The pLacZ control is a Lac promoter–*lacZ* fusion without added IPTG. Average and standard deviation error bars are for *n* = 3 independent replicates. **P > 0.05, *P > 0.1; two-tailed *t*-test. (**F**) Log_2_-fold change in *mtnK* mRNA expression from transcriptomic analysis (Fig. 2; Data set S1) and measured by qRT-PCR for wild-type ATCC 25922 (WT), *mtnR* deletion strain (Δ*mtnR*), and *mtnR* deletion strain complemented with *mtnR* from a plasmid (Δ*mtnR* + pmtnR). Tetracycline (10 ng/mL) was added to induce expression of *mtnR* from pmtnR plasmid. qRT-PCR average and standard deviation error bars are for *n* = 4 independent biological replicates except for WT Glc + 5dR versus Glc (*n* = 8) and Δ*mtnR* + pmtnR Glc + 5dR versus Glc (*n* = 3). **P > 0.05; two-tailed *t*-test.

Using ATCC 25922 as a model, we probed the location of the DHAP shunt promoter(s) using *lacZ* gene fusions with increasingly smaller segments of the DHAP shunt operon 5′-UTR (Fig. 3A). High LacZ activity was observed from the extracts of cells grown with 5dR versus glucose as the sole carbon source (10–16-fold increase) for *lacZ* promoter fusions that included 126 bp or more of the 5′-UTR beginning from *mtnK* (Fig. 3B). Coordinately, a twofold increase in LacZ activity was observed in extracts of cells grown with 5dR plus glucose versus glucose as the sole carbon source, also for *lacZ* promoter fusions that included 126 bp or more of the 5′-UTR. The observed changes in LacZ activity in the reporter assays were verified to be correlated to changes in *lacZ* gene transcript levels based on qRT-PCR of the 255 bp 5′-UTR–*lacZ* fusion construct (pLacZ255; Fig. S3B). Coordinately, the *lacZ* expression levels corroborate the transcriptomics data of wild-type ATCC 25922 growing on glucose versus 5dR (Fig. 2A). In addition, during growth on glucose as the sole carbon source, LacZ activity of strains with DHAP shunt operon 5′-UTR–*lacZ* fusions was the same as for strains with Lac promoter–*lacZ* fusions (pLacZ), indicating that glucose tightly controls expression of the DHAP shunt, like the Lac operon (Fig. 3B; Fig. S4B). Altogether, these results indicate that the promoter(s) for the DHAP shunt resides between 66 and 126 bp upstream of *mtnK*, and the DHAP shunt operon is under control of *E. coli’s* CCR mechanism (see below).

The location(s) of DHAP shunt operon transcription initiation was determined by TSS mapping. cDNA was generated from mRNA of cells grown on glucose, 5dR, or 5dR plus glucose, and TSSs were mapped by primer extension using fluorescently labeled primers and dideoxynucleotide sequencing ladders, as resolved by PAGE. Mapping revealed a primary TSS (P1) located 72 bp upstream of *mtnK* and an alternative TSS (P2) about 188 bp upstream of *mtnK* (Fig. 5B and C). At P1, canonical −10 and −35 elements were identified (Fig. 5A), and qualitatively, mRNA transcript abundance originating from this promoter, based on fluorescence intensity (Fig. 5B), shows the same regulatory pattern as the transcriptomic and LacZ assays. At P2, an AT-rich region consistent with a −10 promoter element is present with no discernible −35 element (Fig. 5A), and qualitative inspection of the mRNA transcript abundance originating from this promoter, based on fluorescence intensity, does not exhibit visible regulation under the tested growth conditions (Fig. 5C). No further TSSs were identified upstream of P2 (Fig. S5B). To confirm P2 is negligibly regulated by glucose versus 5dR availability, three plasmids (placZUR1, placZUR2, and placZUR3) were constructed containing *lac* fusions with distal DHAP shunt 5′-UTR regions that only included or did not include P2 (Fig. 3A). At most, a twofold increase in LacZ activity was observed for fusions of *lacZ* with the P2 promoter when cells were grown on 5dR versus glucose (Fig. 3D), in contrast to the regulation observed from P1. Coordinately in all cases, LacZ activities were less for *lacZ* fusions with the P2 promoter compared to *lacZ* fusions with the P1 promoter for each growth condition (Fig. 3B and D). Thus, P1 with TSS located 72 bp upstream of *mtnK* is the primary and regulated promoter for DHAP shunt expression in response to growth substrate availability. The P2 with TSS located 188 bp upstream is a low-activity secondary promoter whose genetic and physiological relevance is unknown.

### The DHAP shunt is not upregulated during growth on other alternative substrates

**Fig 4:**
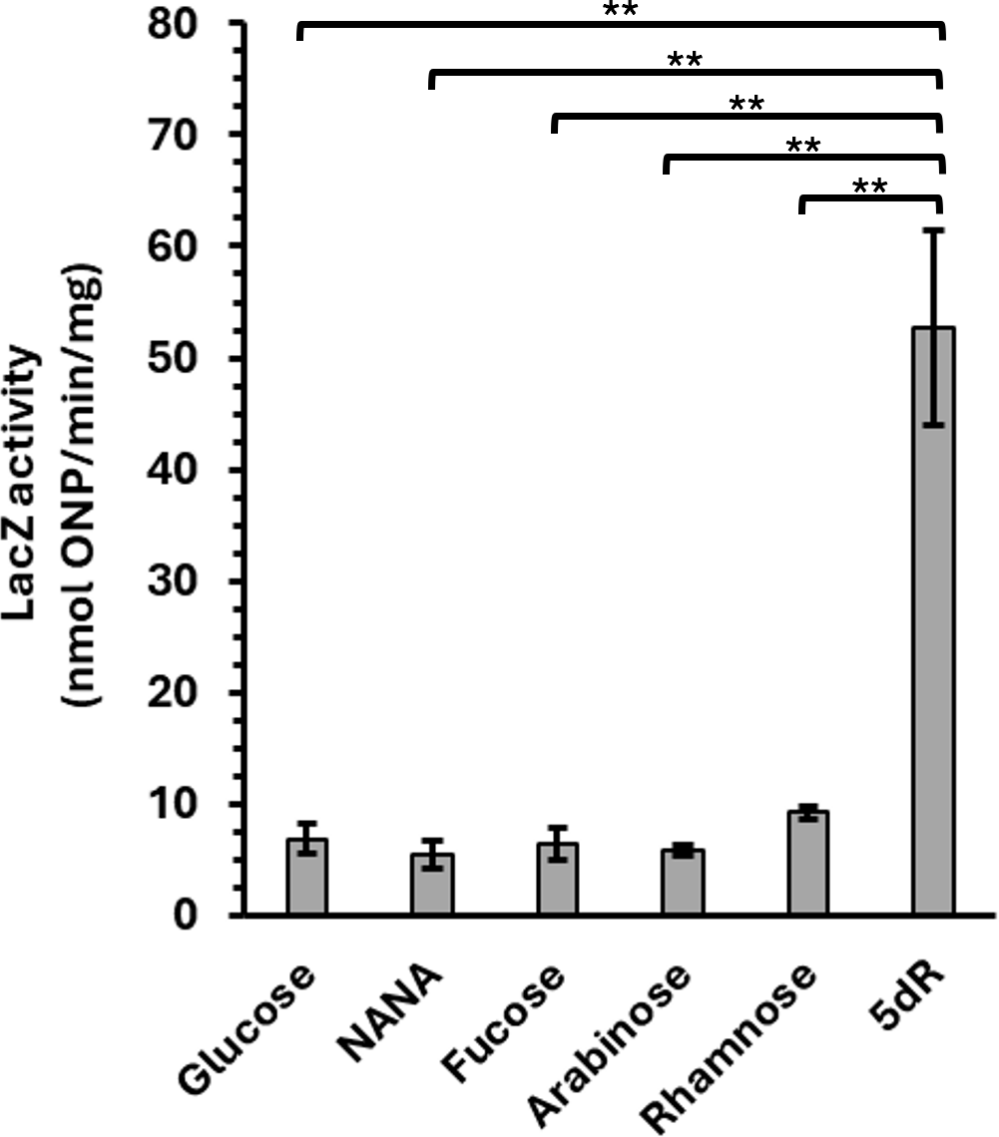
DHAP shunt expression is not activated by other alternative growth substrates. LacZ activity assays from cell extracts of *E. coli* ATCC 25922 containing the DHAP shunt 5′-UTR–*lacZ* fusion plasmid placZ255 and grown with either 5 mM glucose, NANA, l-fucose, l-arabinose, l-rhamnose, or 5-deoxy-d-ribose (5dR) as the sole carbon source. Average and standard deviation error bars are for *n* = 3 independent replicates. **P > 0.05.

To further investigate the impact of substrate identity on DHAP shunt gene expression, we measured DHAP shunt promoter activity using DHAP shunt promoter–*lacZ* fusions (placZ255) (Fig. 3A). Cells were grown on glucose, 5dR, glucose plus 5dR, NANA, fucose, arabinose, and rhamnose. In addition, cells previously grown on glucose were placed under carbon starvation (no growth substrate) conditions for 40 min (Fig. 4; Fig. S3). In contrast to 5dR as the growth substrate, no significant increase was observed in LacZ activity for strains with DHAP shunt promoter–*lacZ* fusions when grown with any of the other alternative substrates tested as the sole carbon source (Fig. 4). Similarly, carbon starvation did not show a significant increase in LacZ activity (Fig. S3A). This corroborates the transcriptomics data indicating that the DHAP shunt is not a pathway generally induced in response to carbon starvation and rather demonstrates that 5dR specifically induces the DHAP shunt operon versus other well-characterized alternative *E. coli* growth substrates (e.g., fucose, NANA, rhamnose). Whether other substrates such as 5dAdo, MTA, and MTR also specifically induce the DHAP shunt operon is a topic for future investigation.

### MtnR is a repressor of DHAP shunt gene expression

Directly downstream and opposing the DHAP shunt operon is an annotated Deoxyribonucleoside Operon Repressor (DeoR) family transcriptional regulator designated as the *yjhU* gene product, which we propose to rename as *mtnR* (Fig. 1A and 3A). The DeoR family of regulators is a known repressor of sugar metabolism pathways, including glycerol-phosphate, ascorbate, and 2′-deoxyribonucleoside metabolism (43). The proximity of an annotated DeoR-family transcriptional regulator gene relative to the DHAP shunt operon suggested potential involvement as a transcriptional regulator of DHAP shunt operon expression.

A comparative transcriptomic analysis between a gene deletion of the DeoR-type transcriptional regulator (∆*mtnR*; *yjhU* deletion strain) and WT ATCC 25922 cultures grown on glucose revealed that the DHAP shunt operon was upregulated (~5 log_2_-fold) in the absence of the DeoR-type transcriptional regulator (Fig. 2B). The observed change in DHAP shunt gene expression between the wild-type and *yjhU* deletion strains grown on glucose suggests that the DeoR-type transcriptional regulator is a repressor of DHAP shunt operon expression. However, the DeoR-type regulator does not appear to be the sole regulator of the DHAP shunt, as DHAP shunt expression levels in the *yjhU* deletion strain grown on glucose were still ~2.5 log_2_-fold less than when the *yjhU* deletion strain was grown on 5dR. This further implicates additional CCR mechanisms in DHAP shunt regulation (see Carbon Catabolite Repression section below). Thus, we propose to designate *yjhU* as *mtnR*, which stands for Methionine Salvage Regulator of *mtnK*, *mtnA*, and ald2 for consistency with established nomenclature where MtnK and MtnA are Methionine Salvage enzymes for metabolism of substrates MTA, MTR, 5dAdo, and 5dR that originate from methionine (i.e., SAM) (13, 44).

To verify the role of MtnR on DHAP shunt gene expression, we conducted LacZ activity assays using the same DHAP shunt promoter–*lacZ* gene fusions as before (Fig. 3A). For both ATCC 25922 wild-type and Δ*mtnR* strains transformed with the promoter–*lacZ* fusion plasmids containing P1 (placZ126 or larger) or P1 and P2 (placZ255 or larger), the same maximal level of LacZ activity was measured in cells grown with 5dR as the sole carbon source (Fig. 3B and C). However, LacZ activity for the Δ*mtnR* reporter strains grown with glucose as the sole carbon source showed a fourfold increase in LacZ activity compared to wild-type strains grown on glucose, such that the LacZ activity for Δ*mtnR* reporter strains grown on glucose was similar to strains grown on 5dR plus glucose (Fig. 3B and C). Furthermore, the repressive effect of MtnR appears specific to P1. No significant difference was observed in LacZ activity for DHAP shunt promoter–*lacZ* fusions only containing P2 (placZUR3) or no known promoter (placZUR1, placZUR2) in the *mtnR* deletion strain (Fig. 3E). We ruled out any unexpected artifacts of using LacZ reporter assays to characterize the regulatory role of MtnR by corroborating these results with direct qRT-PCR analysis of *mtnK* (Fig. 3F). In wild-type ATCC 25922, the log_2_-fold change of *mtnK* transcript in cells grown on 5dR compared to glucose as sole carbon sources was 10.7 ± 0.76, similar to that observed by transcriptomic analysis (~8). Coordinately, deletion of *mtnR* resulted in higher *mtnK* transcript levels when grown on glucose, thus resulting in the ΔmtnR strain grown on 5dR versus on glucose only exhibiting a 1.35 ± 0.57 log_2_-fold difference in *mtnK* transcript levels. This effect is specific to MtnR, as repression was restored upon complementation of Δ*mtnR* with *mtnR* from a plasmid (pMtnR; Fig. 3F).

**Fig 5:**
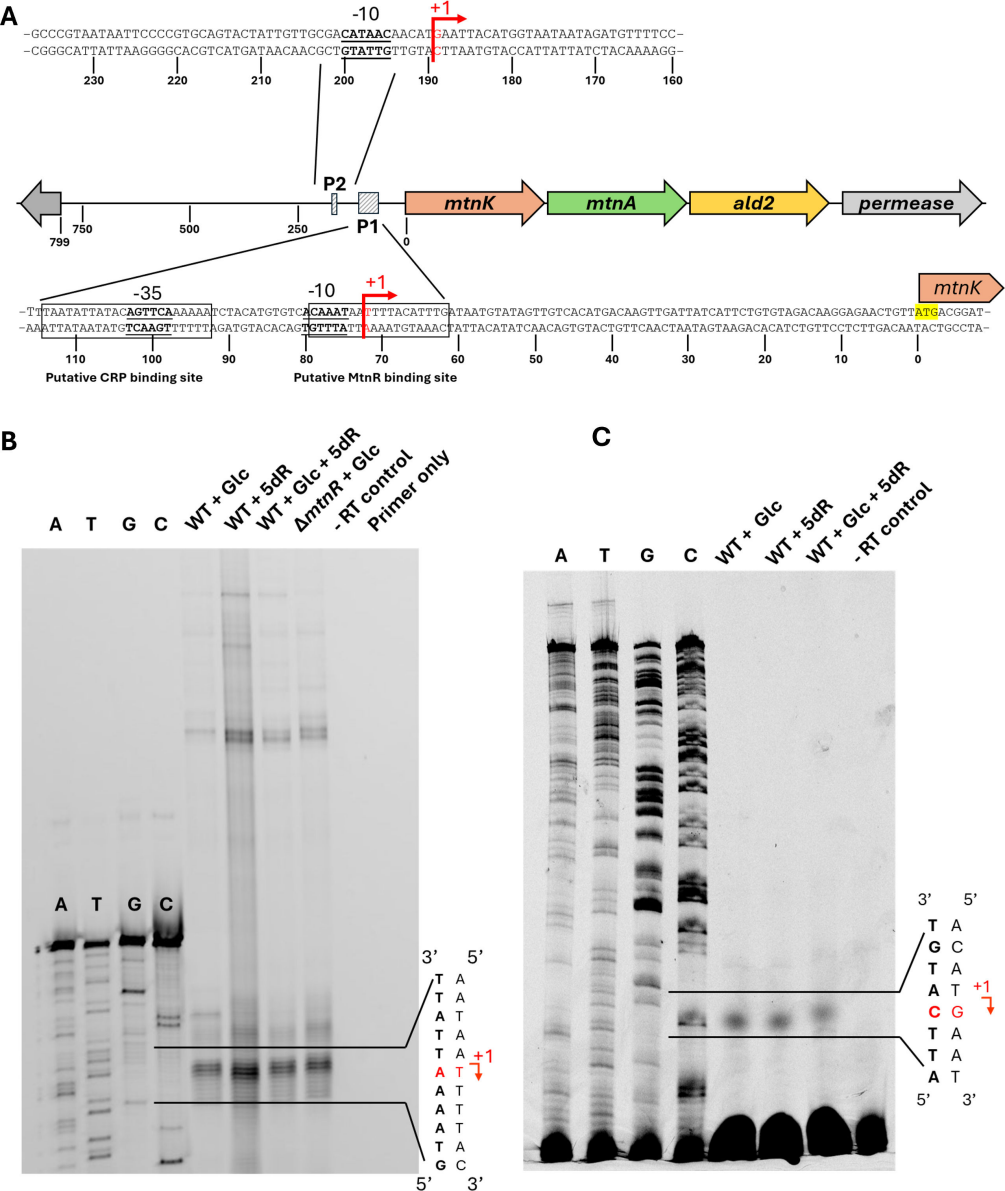
Identification of two TSSs upstream of the DHAP shunt operon. (**A**) Schematic representation of predicted promoters and regulatory elements upstream of *mtnK* based on TSS mapping and CRP binding site (CRPBS Finder) analysis. (B and C) Primer extension mapping of TSSs using two upstream regions: 104 bp upstream of *mtnK* to 45 bp into the gene (**B**), and 142-433 bp upstream of *mtnK* (**C**). Two distinct TSSs are identified: one located 72 bp upstream of *mtnK* (designated P1) and a second 218 bp upstream (designated P2). Based on band intensity, P1 appears to be the primary and regulated promoter under conditions tested. Glc, glucose; 5dR, 5-deoxyribose; −RT control is primer extension of RNA from WT + Glc cells without any added reverse transcriptase; primer only is the primer extension reaction performed without any added RNA.

Taken together, these results support a model in which MtnR is a repressor of the DHAP shunt P1 promoter unless a DHAP shunt substrate like 5dR is available. Currently, the recognition sequence and specific location of MtnR binding for regulation of the DHAP shunt are unknown. However, there is a semi-palindromic sequence (CAAATxxxxxxxxxATTTG) located over the P1 −10 element that may function as the binding site for MtnR or other regulatory mechanisms, and is the focus of future studies (Fig. 5A).

### The DHAP shunt is under the control of *E. coli* carbon catabolite repression

**Fig 6:**
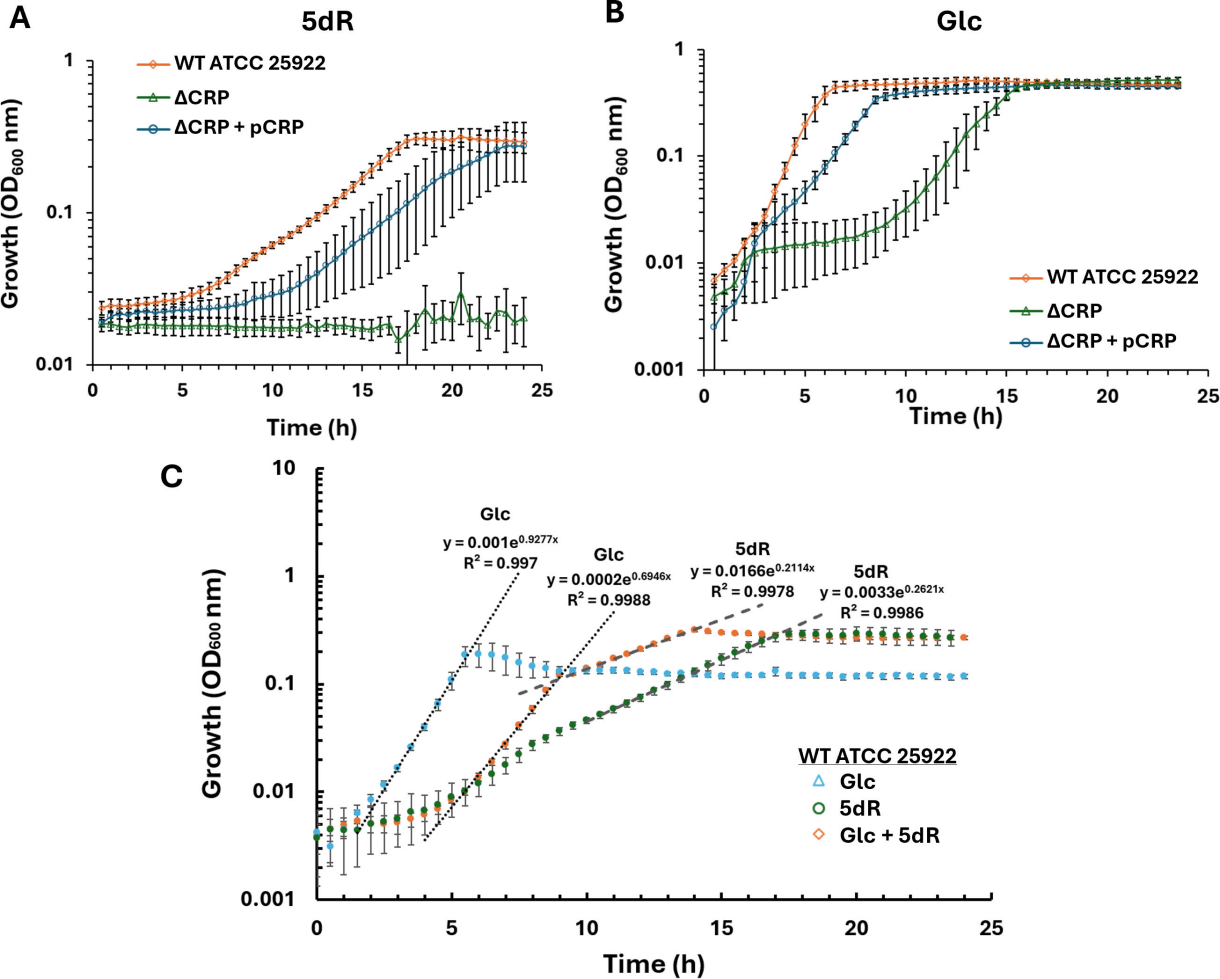
CRP is required for growth of *E. coli* ATCC 25922 using 5dR as a substrate. (**A and B**) Growth of wild-type ATCC 25922 (diamonds), CRP deletion strain (Δ*crp*; triangles), and CRP deletion strain complemented with *crp* expressed from a plasmid (Δ*crp* + pCRP; circles) with (**A**) 5dR or (**B**) glucose as the sole carbon source. Average and standard deviation error bars are for *n* = 4 for growth on 5dR and *n* = 5 for growth on glucose. (**C**) Diauxic growth observed when wild-type ATCC 25922 is grown on a combination of 1 mM glucose plus 8 mM 5dR (diamonds), as compared to cultures grown with 1 mM glucose (triangles) or 8 mM 5dR (circles) as the sole carbon source. Dotted lines and fit parameters are for exponential regression fit to the growth data. Average and standard deviation error bars are for *n* = 5 independent biological replicates.

In many instances where a DeoR transcriptional regulator family member functions as a repressor, the promoter is also subject to activation by another transcriptional regulator (45, 46). In *E. coli*, the glycerol-3-phosphate regulon (specifically *glpD* and *glpE*) and the 2-deoxyribose operon (*deoCBAD*) are negatively regulated by GlpR and DeoR, respectively, and activated by cAMP-bound CRP (45, 46). Previous growth studies have shown that ATCC 25922 is able to grow solely on DHAP shunt pathway substrates, including MTA, 5dAdo, and 5dR (11). To determine if CCR via CRP controls growth on 5dR, a genomic deletion of *crp* in ATCC 25922 (strain ∆*crp*) was constructed. The ∆*crp* strain was able to grow in M9 minimal media with glucose as the sole carbon source, as previously reported for *E. coli* UTI89 (47) (Fig. 6B). However, Δ*crp* was unable to grow on 5dR as the sole carbon source, indicating that the presence of CRP is necessary for growth on 5dR (Fig. 6A). Coordinately, as expected, the *E. coli* ∆*crp* strain could not grow on lactose, NANA, or glycerol, confirming inactivation of CCR in ATCC 25922 (Fig. S6). Complementation of the ∆*crp* strain with *crp* (∆*crp* + pCRP) restored growth to similar rates on 5dR, lactose, NANA, and glycerol (Fig. 6; Fig. S6).

**Fig 7:**
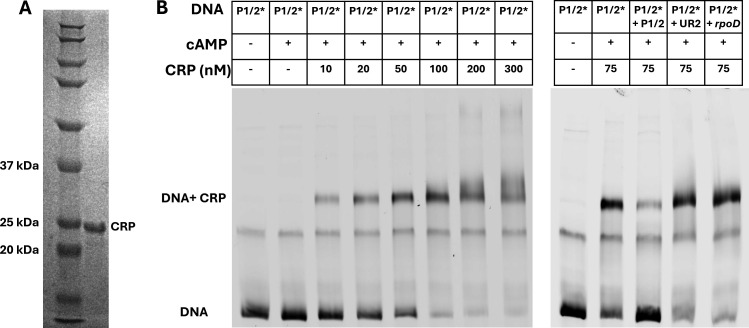
CRP binds to the DHAP shunt promoter. (**A**) Coomassie stained SDS-PAGE gel of N-terminal hexahistidine-tagged CRP purified by Ni-NTA affinity chromatography. (**B**) EMSA of CRP binding to the P1 and P2 DNA fragment (P1/2) which is located within the 255 bp region upstream of *mtnK*. P1/2* indicates 22 nM of a 1:10 ratio of Cy5-labeled:unlabeled P1/2 DNA fragment added to the CRP binding assay. P1/2* + P1/2 indicates an additional 180 nM of unlabeled P1/2 DNA fragment for a 1:100 ratio of labeled:unlabeled and demonstrates exchange of CRP between the labeled and unlabeled P1/2 DNA fragment. P1/2* + UR2 indicates 200 nM of an unlabeled DNA fragment from the region upstream of P1 and P2 as shown in Fig. 3A, and demonstrated CRP does not bind to the genomic region upstream of the DHAP shunt P1 and P2. P1/2* + *rpoD* indicates 200 nM of an unlabeled DNA fragment of the *rpoD* gene, and demonstrated CRP does not bind as a control, similar to UR2.

We confirmed that *E. coli* strain ATCC 25922 preferentially utilizes glucose over the DHAP shunt substrate 5dR through CCR by conducting growth studies of cultures supplemented with glucose plus 5dR, glucose only, and 5dR only (Fig. 6C). Consistent with CCR regulation, growth on glucose plus 5dR resulted in a diauxic growth pattern (Fig. 6C) with a rapid initial growth rate similar to that for glucose only growth (~1 h doubling time), followed by a slower growth rate similar to that for 5dR only growth (~3 h doubling time). To implicate the location of the CRP binding site(s) within the DHAP shunt operon, we analyzed the 250 bp region upstream of the DHAP shunt using CRP Binding Site Finder (CRPBS Finder) software (7). The predictive software identified two potential CRP binding sites, with scores reflective of confidence for CRP interaction. The high-scoring, high-confidence site (6.7 score) is centered at −35.5 position, which is consistent with the organization of characterized class II CRP-dependent promoters where the CRP binding site is positioned near −41.5 (48, 49). The second, lower confidence site (6.1 score) centers at −17.5 (not shown). To verify CRP binding to the DHAP shunt promoter, CRP was purified and binding analyzed by EMSA (Fig. 7). The formation of a single band observed upon complexing of CRP with the P1/2 DNA fragment, followed by hyper-shifts at higher (>200 nM) CRP concentrations consistent with CRP multimers, suggests a single dominant CRP binding site in the DHAP shunt promoter region. This is consistent with the single band observed for CRP binding to the *gal* and *mal* promoter regions and is distinct from the two specific bands observed to form when CRP binds to the CRP1 and CRP2 sites of the *lac* promoter centered at −61.5 and +11.5, respectively (50, 51). Notably for the *lac* promoter, the −61.5 site is the primary high-affinity site for activation, and the low-affinity +11.5 site is predicted to be antagonistic at higher CRP concentrations (50, 52). While we cannot conclusively rule out CPR binding to the predicted −17.5 site of the DHAP shunt P1 promoter (i.e. this complex may be unstable during EMSA), if CRP were to bind here, it would also likely act as an antagonist to the primary CRP binding site near −35.5 for transcriptional activation (52). The precise location of CRP binding and affinity to the DHAP shunt promoter are a focus of future studies. Altogether, these results demonstrate that CRP is involved in the activation of DHAP shunt expression and that *E. coli* has placed the DHAP shunt under its CCR hierarchy for the use of 5-deoxynucleosides and 5-deoxypentose sugars after glucose becomes limiting.

## DISCUSSION

*E. coli* encodes a diverse range of metabolic pathways, particularly those involved in carbon metabolism that aid cellular survival in dynamic environments. *E. coli* strains with the DHAP shunt enable them to use extracellular 5′-deoxynucleosides and 5-deoxypentose sugars as sole carbon and energy sources, save for fermentative growth (11, 12). This work reveals that *E. coli* ATCC 25922 has placed the DHAP shunt under its hierarchical regulation of carbon metabolism pathways to first use preferential substrates like glucose and gluconate before turning to a 5-deoxypentose sugar like 5dR as a growth substrate. Based on the comparative sequence analysis of the DHAP shunt promoter, it is anticipated that ExPEC strains with the DHAP shunt, like those of the globally disseminated ST131 lineage, do the same (Fig. 1B). Regulation of the DHAP shunt operon is mediated by two transcriptional regulators: CRP, functioning as a transcriptional activator that binds to the DHAP shunt promoter when glucose is absent (Fig. 6 and 7), and MtnR, acting as a transcriptional repressor when 5dR is absent (Fig. 2 and 3). Although identification of allosteric regulators of MtnR and the specific interactions between each regulator and the operon’s promoter require further investigation, identification of a canonical −10 and −35 promoter region designated P1 with TSS located 72 bp upstream of *mtnK* facilitates the prediction of putative CRP and MtnR binding sites (Fig. 5A). The predicted CRP binding site centered at −35.5 upstream of *mtnK* is anticipated to be part of a class II CRP-dependent promoter, similar to the characterized *galP1* and *pmelR* Class II CRP-dependent promoters of *E. coli* with CRP binding sites centered at −41.5 (49). In general, class II binding sites are associated with activation of downstream genes (48). At class II binding sites, CRP occludes the −35 element from recognition by the sigma factor within the RNA polymerase holoenzyme (53). Instead, domains of the α-subunit of RNA polymerase interact directly with CRP to mediate polymerase recruitment and subsequent activation, independent of the −35 element (49).

Regarding MtnR, the DeoR family of transcriptional regulators to which it belongs regulates the expression of numerous genes, including those involved in carbon metabolism pathways (43). In *E. coli*, DeoR family regulators control the expression of genes for glycerol (GlpR), fucose (FucR), and 2´-deoxyribonucleoside (DeoR) metabolism (43). These repressors are regulated by allosteric effector molecules, typically a phosphorylated pathway intermediate (e.g., sn-glycerol-3-phosphate, fuculose-1-phosphate, 2-deoxyribose-5-phosphate), which modulates DeoR family regulator binding affinity to its DNA recognition sequence (46). For DHAP shunt regulation by MtnR, the semi-palindromic sequence observed by visual inspection to overlap the P1 promoter −10 element is a hallmark of many DeoR family repressors (46). Analogous to the binding of DeoR to its recognition site over the −10 element of the promoter for the 2-deoxyribose metabolism operon (*deoCABD*), anticipated occupation of the DHAP shunt operon −10 element by MtnR would sterically hinder RNA polymerase binding or translocation, thereby repressing transcription initiation as observed in this study (Fig. 3 and 5). Binding of an allosteric effector molecule, potentially 5-deoxyribose-1-phosphate or some other phosphorylated intermediate of the DHAP shunt, would cause MtnR to release from the DNA. Together, the dual regulation by CRP and MtnR underscores the sensitivity of the DHAP shunt gene expression to substrate availability as well as further supports the pathway’s identity as an alternative carbon metabolism pathway where multiple regulatory factors fine-tune gene expression (54).

Unlike the DHAP shunt gene cluster, which is limited to select ExPEC strains (12), *mtnR* (annotated in *E. coli* genomes as *yjhU*) is widespread across *E. coli*. This protein’s putative function was predicted as a transcription factor, yet a clear regulatory function has remained elusive (55–58). In *E. coli* K12 strains lacking the DHAP shunt, *mtnR* is observed to be upregulated in stress response conditions, such as the presence of volatile organic compounds, and *mtnR* expression correlates with expression of stress response regulatory genes *soxS* and *rpoS*, suggesting *mtnR* expression may be controlled by a master regulator (59). In ExPEC ST131, MtnR has also been shown to increase in abundance in relation to outer membrane vesicles during iron limitation (60). Here, we demonstrated that MtnR has a significant repressive effect on DHAP shunt gene expression. Furthermore, deletion of *mtnR* resulted in upregulation of not only the DHAP shunt genes, but also *efeU* (DR76_RS21025, Fig. 2B), a gene encoding an iron permease induced under iron-limiting and acidic conditions (61, 62). For effective iron uptake by the Efe system, expression of the *efeUOB* operon genes is required and is known to be regulated by Fur and CpxR (61–63). Based on this study and previous work (60), it appears that MtnR is also involved in the expression of *efeUOB* in *E. coli*. In *E. coli*, it is common for transcriptional regulators to modulate expression of multiple targets (64), and within the DeoR transcriptional regulator family, there are examples of members that repress multiple targets (43, 64). Thus, these observations indicated that *mtnR* is conserved across *E. coli* strains for the regulation of conserved pathways like *efeUOB*, even though the DHAP shunt is only primarily found in ExPEC strains.

ExPEC are pathogenic strains of *E. coli* that are responsible for infections outside of the gastrointestinal tract, including the bloodstream, cerebral fluid, and urinary tract (17). These niches are generally considered nutrient-limiting, with carbon and sulfur sources either scarce or present in compounds not typically utilized as nutrients (10, 65). Given the ubiquity of SAM in all domains of life, the SAM reaction byproducts—SAH, MTA, and 5dAdo—are also widespread. While SAH is recycled to methionine (13), MTA and 5dAdo, or their downstream products, are generally exported from cells as metabolic waste (13). In prokaryotes, MTA/5dAdo phosphorylase (MtnP) or MTA/5dAdo/SAH nucleosidase (MtnN, Pfs) processes MTA and 5dAdo into 5-deoxypentose-phosphate species or 5-deoxypentose species, respectively, prior to excretion (13, 65). Eukaryotes only use MtnP (66). Notably, in extraintestinal environments such as the urine, these compounds are observed to accumulate (~0.1 µmol/mmol creatinine or ~100 µM) compared to the intestine (22). The current paradigm of ExPEC colonization of the urinary tract centers around metabolism of proteogenic and non-proteogenic amino acids as primary carbon and nitrogen sources, which occur at 10–30 µmol/mmol creatinine (10, 22). Therefore, further work is required to determine the contribution of 5-deoxynucleosides and 5-deoxypentose sugars to the growth of ExPEC strains with the DHAP shunt compared to amino acids and other alternative growth substrates.

## Data Availability

All strains, plasmids, and source data will be made available upon request. Transcriptome datasets for E. coli ATCC 25922 wild type and mtnR deletions strains are available from NCBI Gene Expression Omnibus (GEO) https://www.ncbi.nlm.nih.gov/geo/ under accession number GSE308804.

## SUPPLEMENTAL MATERIAL

Dataset S1 jb.00280-25-s0001.xlsx RNAseq transcriptomics gene fold-change values.

Dataset S2 jb.00280-25-s0002.xlsx DHAP shunt alignment and percent coverage.

Dataset S3 jb.00280-25-s0003.txt DHAP shunt and promoter sequences from *E. coli* genomes.

Supplemental materials jb.00280-25-s0004.pdf Supplemental methods, Figures S1 to S6, Tables S1 to S3, Legends for Dataset S1 to S3, and References.

ASM does not own the copyrights to Supplemental Material that may be linked to, or accessed through, an article. The authors have granted ASM a non-exclusive, world-wide license to publish the Supplemental Material files. Please contact the corresponding author directly for reuse.
