# Supplementary material for: Escherichia coli is poised to grow using 5′-deoxynucleosides via MtnR and CRP regulation of DHAP shunt gene expression
Source: J Bacteriol. 2025 Oct 16;207(11):e00280-25. doi: 10.1128/jb.00280-25 (PMC12632272; doi:10.1128/jb.00280-25)
Supplement: Supplemental materials — Supplemental methods, Figures S1 to S6, Tables S1 to S3, Legends for Dataset S1 to S3, and References. [file jb.00280-25-s0004.pdf]

Supplemental Information:

Escherichia coli are poised to grow using 5'-deoxynucleosides via MtnR and CRP regulation of DHAP shunt gene expression

Katherine A. Huening[1*], Caitlin C. Wingerd[1*], Joshua T. Groves[1], Katelyn T. Kapusta[1], Laiba Khan[1], F. Robert Tabita[1], Justin A. North[1#]

1 Department of Microbiology, The Ohio State University, Columbus, OH

* Authors contributed equally to this work

# Corresponding Author: north.62@osu.edu

Inventory of Supplemental Information

Supplemental methods 2

Python Code used in this study 4

Supplementary Figures 1-6 9

Supplementary Tables 1-3 16

Supplementary Dataset Captions 22

Supplementary References 23

Supplementary Methods

***Plasmid construction* –** Native *lacZ* from ATCC 25922 was PCR amplified using primers lacZ-SpeI_F and lacZ-AgeI_R (Supplementary Table 1), which introduced flanking SpeI and AgeI restriction sites. The resulting fragment was cloned into pBBRsm2-MCS6 [1], replacing the plasmid's *lacZα* gene within the multiple cloning site. This resulted in the plasmid placZ, which has *lacZ* under regulatory control of the IPTG-inducible Lac promoter containing the Lac operator and CRP binding site. Next, plasmids placZ799 and placZ255 were constructed by PCR amplification of the region directly upstream of ATCC 25922 *mtnK* start codon (either 799 nucleotides or 255 nucleotides) and cloning it directly in front of the *lacZ* gene of placZ using restriction sites AseI and SpeI. The introduced SpeI site was located three bases immediately upstream of the *mtnK* start codon by replacing ACTGTT with ACTAGT. To construct the remaining plasmids used in LacZ assays, placZ799 was used as template for PCR amplification of smaller upstream regions. To construct plasmids placZ169, placZ126, and placZ66, a construct-specific primer, UPS169-AseI_F, UPS126-AseI_F, and UPS66-AseI_F, respectively, was used in combination with primer UPS-BspEI-R, which anneals within the *lacZ* sequence, for PCR amplification (Supplementary Table 1). The PCR products had flanking AseI and BspEI recognition sequences, which were used to clone these fragments into placZ. To construct plasmids placZUR1, placZUR2, and placZUR3, a forward primer UPS799-AseI_F was used in combination with a construct-specific reverse primer, UR1-SpeI_R, UR2-SpeI_R, and UR3-SpeI_R (Supplementary Table 1), respectively, to amplify the far upstream region of the DHAP shunt genes (Figure 3A). To ensure that a ribosome binding site (RBS) was present in these sequences, an RBS sequence (5'-AGGAGA, the same RBS sequence present before *mtnK*) was included in each of these three reverse primers immediately following the SpeI restriction site sequence. To amplify this upstream region, p799 was used as the DNA template. The amplified region was then cloned into a placZ construct using AseI and SpeI restriction sites.

***LacZ activity assays* -** Cells either placZ, placZ799, placZ255, placZ169, placZ126, placZ66, placZUR1, placZUR2, or placZUR3 (Supplementary Table 1) were harvested by centrifugation at 5000 x g for 5 minutes, and cell extracts were prepared by sonication and assayed as described previously [2, 3] with the following changes: the cell pellet was resuspended in 500 μL sonication buffer [25 mM Tris HCl (pH 8.0), 5 mM β-mercaptoethanol] and sonicated on ice for 5 second

pulses over 1.5 minutes. Cell lysates were centrifuged at 18,000 x g for 10 minutes at 4°C. The supernatant was retained; an aliquot was separated to be used in protein concentration quantification by Bicinchoninic Acid (BCA) assay per manufacturer's instructions (Pierce). EDTA was added to the remaining supernatant to a concentration of 1 mM for use in LacZ activity assays. To perform LacZ activity assays, supernatant and Z buffer [50 mM Na-PO_4 buffer (pH 7.0), 10 mM KCl, 1 mM $MgSO_4$, 2.5 mM β-mercaptoethanol] were mixed in a cuvette in a total volume of 500 μL. The LacZ activity assay was initiated with the addition of 100 μL 4 mg/ml *o*-nitrophenyl-β-d-galactopyranoside (ONPG) in 50 mM Na-PO_4 (pH 7.0). Assays were performed at 37°C and changes in absorbance at 420 nm due to the formation of *o*-nitrophenol (ONP) were monitored using a Cary 4000 UV-vis spectrophotometer (Agilent). Activity was calculated using the *o*-nitrophenyl (ONP) molar extinction coefficient of 2,388 M^{-1} cm^{-1} determined at pH 7.0 [4].

CPR Purification - Hexahistidine-tagged *E. coli* CRP proteins were produced in *E. coli* BL21 (DE3) carrying the expression plasmid pET28-crp similar to procedures previously described [5]. Cells were initially growth at 37 °C, 250 rpm shaking until an $OD_{600\,nm}$ of 0.6. CRP expression was then induced with 0.2 mM IPTG and cells were incubated at 18 °C, 200 R.P.M. shaking overnight. Cells were pelleted by centrifugation and cell pellets stored at -80 °C until further use. For purification cells were thawed and resuspended in ice cold Buffer A (50 mM Tris pH 7.5, 300 mM NaCl) with 10 mM imidazole, 2 mM phenylmethylsulfanyl fluoride (PMSF), 2 mM dithiothreitol (DTT), and 1 mM ethylenediaminetetraacetic acid (EDTA). Cells were lysed by French pressure cell and centrifuged at 20,000 x g for 20 minutes at 4 °C. The soluble supernatant was supplemented with 1 mM $MgCl_2$ to chelate the excess EDTA and applied to a Ni-NTA column equilibrated with Buffer A + 10 mM imidazole. The column was washed with 10 column volumes (CV) Buffer A + 10 mM imidazole, followed by washing with 10 CV Buffer A + 20 mM imidazole, and elution with Buffer A + 250 mM imidazole. Purified protein was analyzed by SDS-PAGE and concentrated into TED300 buffer (20 mM Tris pH 7.5, 300 mM NaCl, 1 mM DTT, 1 mM EDTA) by 30 kDa molecular weight cutoff centrifugal concentration device (Millipore). Protein was stored on ice and used within 1 week for EMSA.

Python code used for sequence alignment and visualization:

```
# === INPUT FILES ===
subject_labels_file = "subject_label.txt"  # one label per line (partial matches allowed)
genome_fasta = "corrected_sequences.fasta"
reference_fasta = "ATCC_25922_reference.fasta"

# === LOAD SUBJECT LABELS ===
with open(subject_labels_file) as f:
    subject_labels = [line.strip() for line in f if line.strip()]
print(f"Loaded {len(subject_labels)} subject labels.")

# === LOAD GENOME FASTA SEQUENCES ===
print("Loading genome fasta...")
genome_records = list(SeqIO.parse(genome_fasta, "fasta"))
print(f"Loaded {len(genome_records)} sequences from fasta.")

# === LOAD REFERENCE SEQUENCE ===
ref_record = next(SeqIO.parse(reference_fasta, "fasta"))
ref_seq = str(ref_record.seq).upper()
ref_len = len(ref_seq)
print(f"Reference length: {ref_len}")

# === FUNCTION: Get matching genome record by partial label match ===
def find_matching_record(label, records):
    label = label.lower()
    for rec in records:
        if label in rec.id.lower():
            return rec
    return None

# === FUNCTION: Convert Biopython Alignment object to aligned sequences with gaps ===
def get_aligned_seqs(alignment, seqA, seqB):
    aligned_seqA = []
    aligned_seqB = []
    blocksA = alignment.aligned[0]
    blocksB = alignment.aligned[1]

    posA = 0
    posB = 0

    for (startA, endA), (startB, endB) in zip(blocksA, blocksB):
        while posA < startA:
            aligned_seqA.append(seqA[posA])
            aligned_seqB.append('-')
            posA += 1
```

```
        while posB < startB:
            aligned_seqA.append('-')
            aligned_seqB.append(seqB[posB])
            posB += 1
        aligned_seqA.extend(seqA[startA:endA])
        aligned_seqB.extend(seqB[startB:endB])
        posA = endA
        posB = endB

    while posA < len(seqA):
        aligned_seqA.append(seqA[posA])
        aligned_seqB.append('-')
        posA += 1
    while posB < len(seqB):
        aligned_seqA.append('-')
        aligned_seqB.append(seqB[posB])
        posB += 1

    return ''.join(aligned_seqA), ''.join(aligned_seqB)

# === ALIGNER SETUP ===
aligner = PairwiseAligner()
aligner.mode = 'local'
aligner.match_score = 2
aligner.mismatch_score = -1
aligner.open_gap_score = -0.5
aligner.extend_gap_score = -0.1

# === STORE ALIGNMENTS ===
aligned_subjects = []
aligned_sub_seqs = []
aligned_ref_seqs = []

print("Starting alignment and conservation calculation...")

for label in subject_labels:
    record = find_matching_record(label, genome_records)
    if record is None:
        print(f"Warning: Label '{label}' not found in FASTA.")
        continue

    sub_seq = str(record.seq).upper()
    if len(sub_seq) == 0:
        print(f"Warning: Sequence for '{label}' is empty.")
        continue

    alignment = aligner.align(ref_seq, sub_seq)[0]  # best alignment
    aligned_ref_seq, aligned_sub_seq = get_aligned_seqs(alignment, ref_seq, sub_seq)
```

```
    aligned_subjects.append(label)
    aligned_ref_seqs.append(aligned_ref_seq)
    aligned_sub_seqs.append(aligned_sub_seq)

if not aligned_sub_seqs:
    print("No sequences aligned successfully. Exiting.")
    exit()

# === MATCH/CONSERVATION MATRIX ===
print("Building match matrix for conservation visualization...")
match_matrix = []  # rows: subjects, columns: match (1) or mismatch (0)

for aligned_ref_seq, aligned_sub_seq in zip(aligned_ref_seqs, aligned_sub_seqs):
    match_row = []
    for ref_base, sub_base in zip(aligned_ref_seq, aligned_sub_seq):
        if ref_base == '-' or sub_base == '-':
            match_row.append(None)
        elif ref_base == sub_base:
            match_row.append(1)
        else:
            match_row.append(0)
    match_matrix.append(match_row)

# === PLOT CONSERVATION TRACK WITH COLOR ===
print("Generating alignment visualization...")
fig, ax = plt.subplots(figsize=(18, 8))

for i, row in enumerate(match_matrix):
    for j, val in enumerate(row):
        if val is None:
            continue
        color = 'silver' if val == 1 else 'red'
        ax.add_patch(mpatches.Rectangle((j, len(match_matrix) - i - 1), 1, 1, color=color))

ax.set_xlim(0, len(match_matrix[0]))
ax.set_ylim(0, len(match_matrix))
ax.set_xlabel("Reference Position")
ax.set_ylabel("Subject")
ax.set_title("Alignment Match to Reference (silver = Match, Red = Mismatch)")
ax.set_yticks([])
plt.tight_layout()
plt.savefig("alignment_conservation_colored.png", dpi=300)
plt.show()
print("Saved alignment image as 'alignment_conservation_colored2.png'.")
```

Python code used for percent coverage calculation and visualization:

```
#=== Load data from Excel ===
df = pd.read_excel("Promoter_hits_start_stop_positions.xlsx")

# ===Standardize column names===
df.columns = df.columns.str.lower()

# ===Validate required columns===
required_cols = {'label', 'start', 'end'}
if not required_cols.issubset(df.columns):
    raise ValueError(f"Excel file must contain the columns: {required_cols}")

ref_len = 799

# ===Calculate coverage info===
df['length'] = df['end'] - df['start'] + 1
df['percent_coverage'] = (df['length'] / ref_len * 100).round(2)

# ===Group identical (percent_coverage, start, end)===
grouped = defaultdict(list)
for _, row in df.iterrows():
    key = (row['percent_coverage'], row['start'], row['end'])
    grouped[key].append(row['label'])

# ===Sort groups by percent coverage descending===
sorted_groups = sorted(grouped.items(), key=lambda x: x[0][0], reverse=True)

# ===Prepare data for plotting===
labels = []
bars = []       # bar length = length in bases
positions = []   # bar left = start position

for (percent, start, end), label_group in sorted_groups:
    labels.append(f"{percent}% (n={len(label_group)})")
    bars.append(end - start + 1)  # length in bases, NOT percent
    positions.append(start)       # start position in bases

# ===Plot===
fig, ax = plt.subplots(figsize=(15, max(6, len(labels) * 0.5)))
y_pos = np.arange(len(labels))

ax.barh(y_pos, bars, left=positions, color='skyblue', edgecolor='black')

# ===Set Y-axis Configuration===
ax.set_yticks(y_pos)
```

```
ax.set_yticklabels(labels, fontsize=6)
ax.invert_yaxis()

# ===Set X-axis Configuraiton===
ax.set_xlim(0, ref_len)
ax.set_xlabel('Reference Position (1 to 799)', fontsize=10)
ax.tick_params(axis='x', labelsize=8)

xticks = np.linspace(0, ref_len, num=17).astype(int)
ax.set_xticks(xticks)
ax.set_xticklabels([str(x) for x in xticks], fontsize=6)

ax.set_title('Sequence Coverage Groups Aligned to Reference', fontsize=12)
plt.tight_layout()
plt.show()

# ===Save CSV===
output_filename = "coverage_groups_percent_sorted.csv"
with open(output_filename, "w", newline="") as f:
    import csv
    writer = csv.writer(f)
    writer.writerow(["Percent_Coverage", "Start", "End", "Num_Sequences", "Labels"])
    for (percent, start, end), label_group in sorted_groups:
        writer.writerow([percent, start, end, len(label_group), ";".join(label_group)])

print(f"CSV written to: {output_filename}")
```

Supplementary Figures

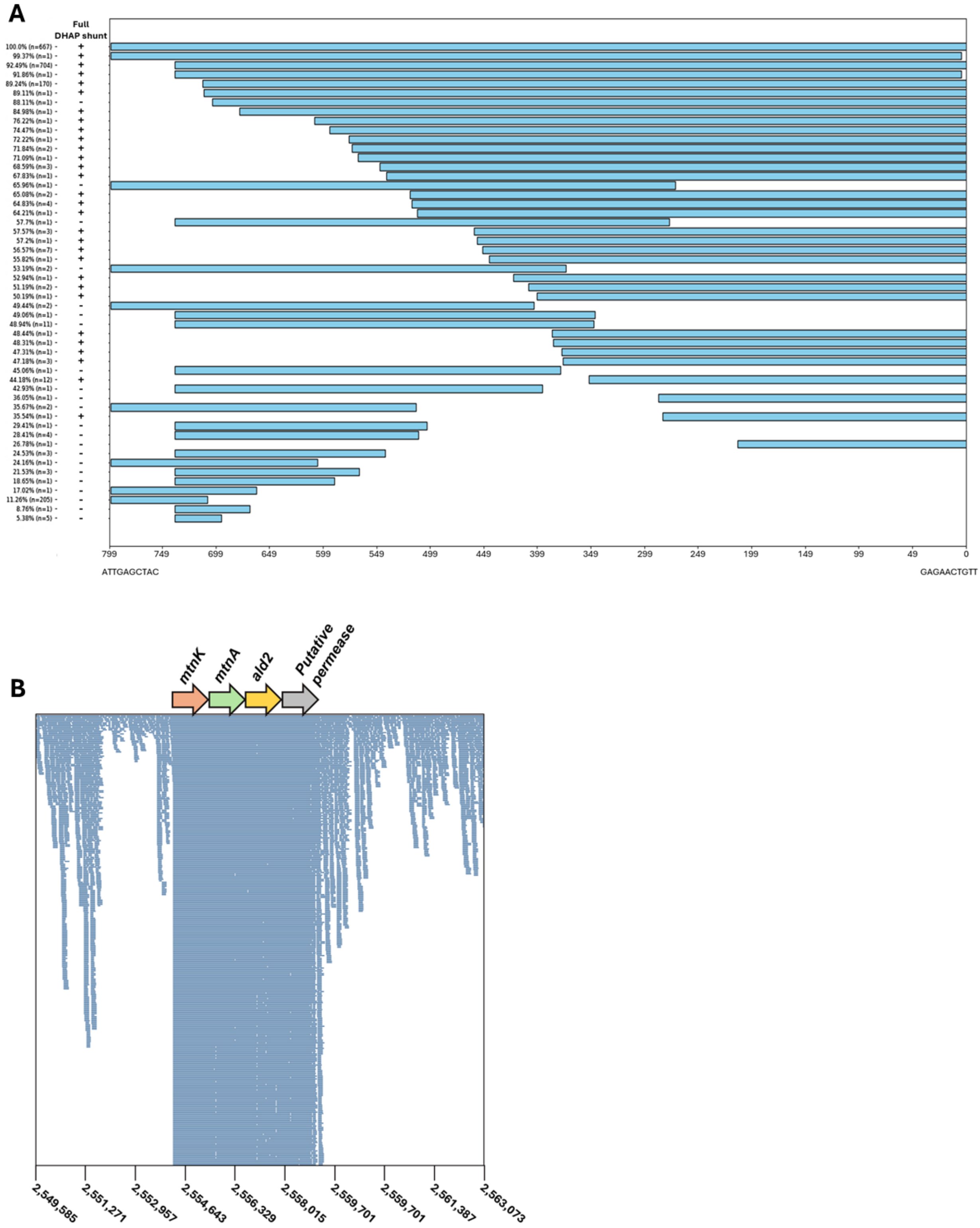

Suppl. Fig. S1. Comparative analysis of upstream region and DHAP shunt operon transcriptional output. A) Alignment and grouping by % coverage of 1,847 *E. coli* genomes with sequence homology to the 799 bp 5'UTR of the ATCC 25922 DHAP shunt operon. The n= values next to each % coverage group is the number of strains with the observed conserved region. Multiple groups with low % coverage were associated with a truncated or no DHAP shunt ("-"). Only strains with > 250 bp of sequence conserved with the ATCC25922 DHAP shunt 5'UTR immediately upstream of mtnK were observed to possess the full DHAP shunt operon ("+"). Only strains with a full DHAP shunt were used in the promoter conservation analysis shown in Figure 1B. **B)** Transcriptome read depth in the region of the DHAP shunt gene cluster. Visual inspection of the transcriptome read depth using Tablet [6] shows similar reads from each of the DHAP shunt genes, suggesting this cluster is transcribed as a polycistronic message and thus a DHAP shunt operon.

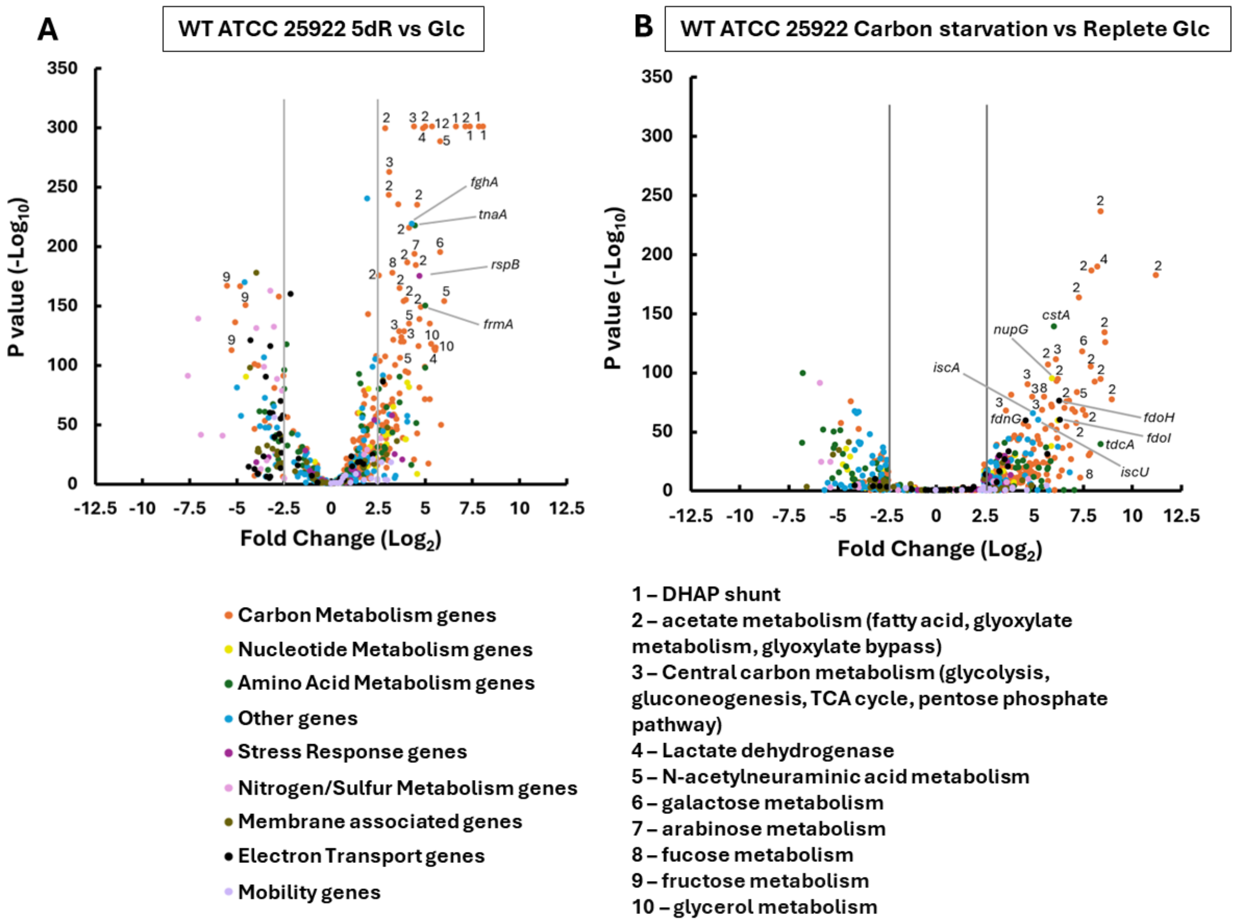

Suppl. Fig. S2. Comparison of ATCC 25922 transcriptome changes based on gene functional annotation. Volcano plots of changes in ATCC 25922 transcript abundance for: **A)** Wild type ATCC 25922 grown with 5dR versus glucose, **B)** ATCC 25922 incubated under carbon starvation conditions versus carbon replete (glucose) conditions. Gray lines indicate 2.5 $\log_2$-fold change thresholds. A total of 664 annotated genes with ≥2.5 $\log_2$-fold change in either condition was included; genes lacking annotation or with unknown function were excluded. Gene categorization is based on UniProt and NCBI functional annotations. The two transcriptional profiles indicate distinct cellular responses: carbon starvation versus carbon replete (glucose) conditions broadly upregulates alternative carbon metabolism pathways, while growth on 5dR versus glucose elicits a more specific response.

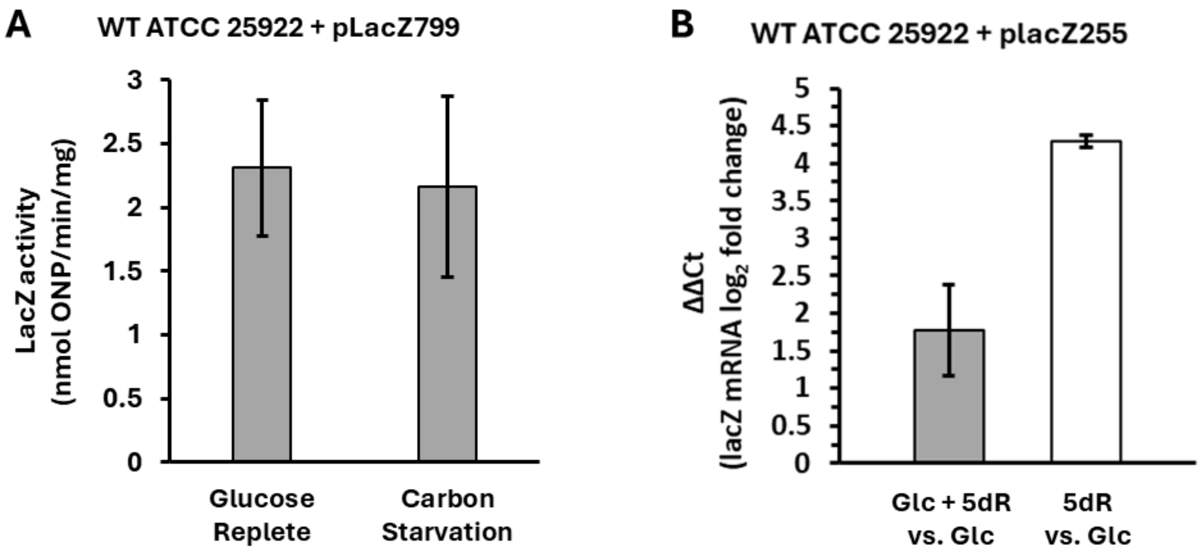

Suppl. Fig. S3. DHAP shunt genes are under the regulatory influence of MtnR, but not as a starvation response. **A)** LacZ activity assays from cell extracts of glucose starved and glucose replete *E. coli* ATCC 25922 containing the DHAP shunt 5'UTR - *lacZ* fusion plasmid placZ799. Cells were grown with 5 mM glucose as the sole carbon source. Once cultures reached midlog phase, cells were washed with carbon free M9 media, then half of the culture was supplemented with 5 mM glucose and the other half remained without. Cultures were further incubated at 37°C, 250 rpm, for an additional 40 minutes before harvesting for LacZ assays. Average and standard deviation error bars are for n = 3 independent replicates. **B)** Fold change in *lacZ* mRNA expression from the DHAP shunt 5'UTR - *lacZ* fusion plasmid placZ255 in *E. coli* ATCC 25922 measured by qRT-PCR. Cultures were grown in M9 minimal medium with 5 mM glucose (Glc), 5 mM glucose + 5 mM 5-deoxy-D-ribose (Glc + 5dR) or 5 mM 5dR. Sigma factor gene *rpoD* was used as a reference gene. Average and standard deviation error bars are for n = 4 independent replicates except for Glc + 5dR cultures, in which n = 3.

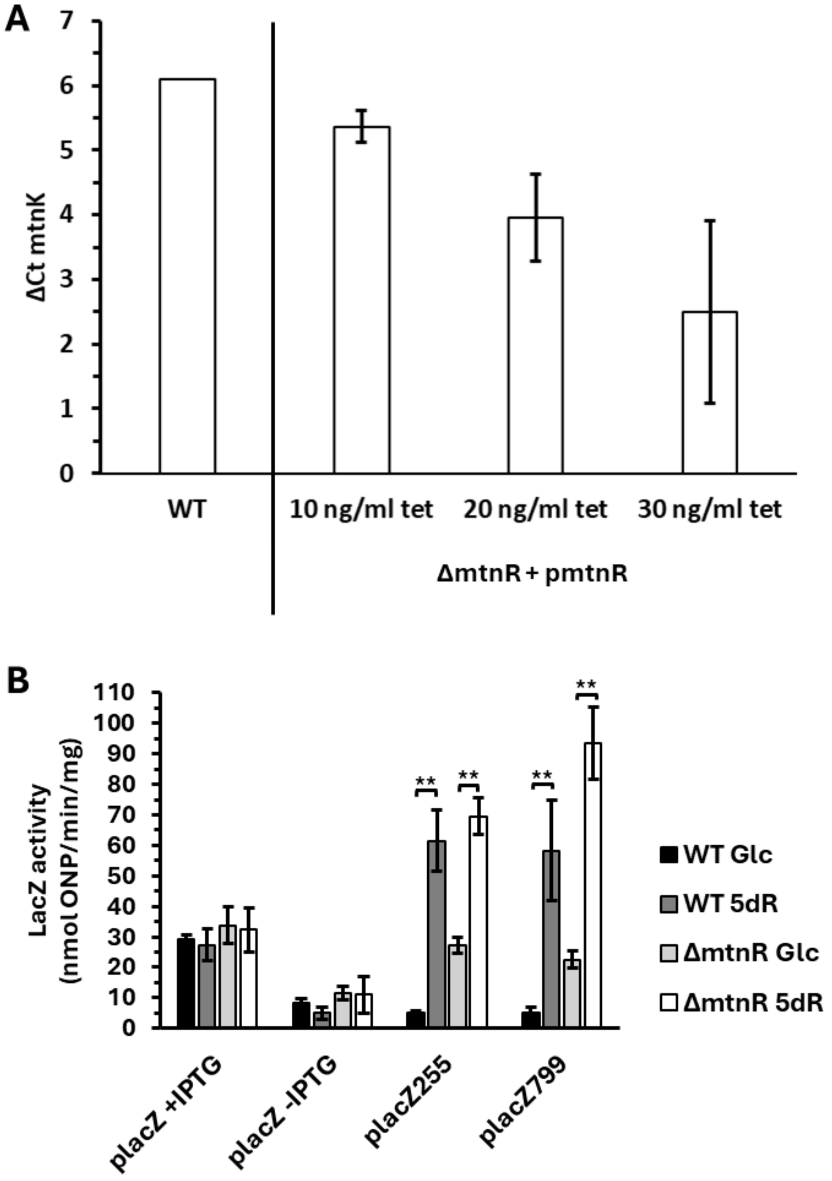

Suppl. Fig. S4. A) Changes in ΔCt values for *mtnK* transcript relative to *rpoD* transcript measured by qRT-PCR for wild type ATCC 25922 (WT) and MtnR deletion strain complemented with *mtnR* expressed from a plasmid (Δ*mtnR* + pmtnR). Complementation studies were initially performed under different concentrations of tetracycline to determine the concentration at which *mtnK* was expressed in the complementation strain at levels similar to WT. Growth with 50 ng/ml tetracycline and higher (not shown) resulted in stressed cells and poor growth. **B)** LacZ activity assays from cell extracts of wild type *E. coli* ATCC 25922 (WT) and ATCC 25922 MtnR deletion strain (Δ*mtnR*) containing either placZ or the DHAP shunt 5'UTR - *lacZ* fusion plasmids, placZ255 or placZ799 (Figure 3A). Cells were grown with either 25 mM glucose (Glc) or 5 mM 5-deoxy-D-ribose (5dR) as the sole carbon source. No IPTG was added to the placZ – IPTG cultures, 0.2 mM IPTG was added to the placZ +IPTG cultures. Average and standard deviation error bars are for n = 3 independent replicates, **$P > 0.05$; *$P > 0.1$.

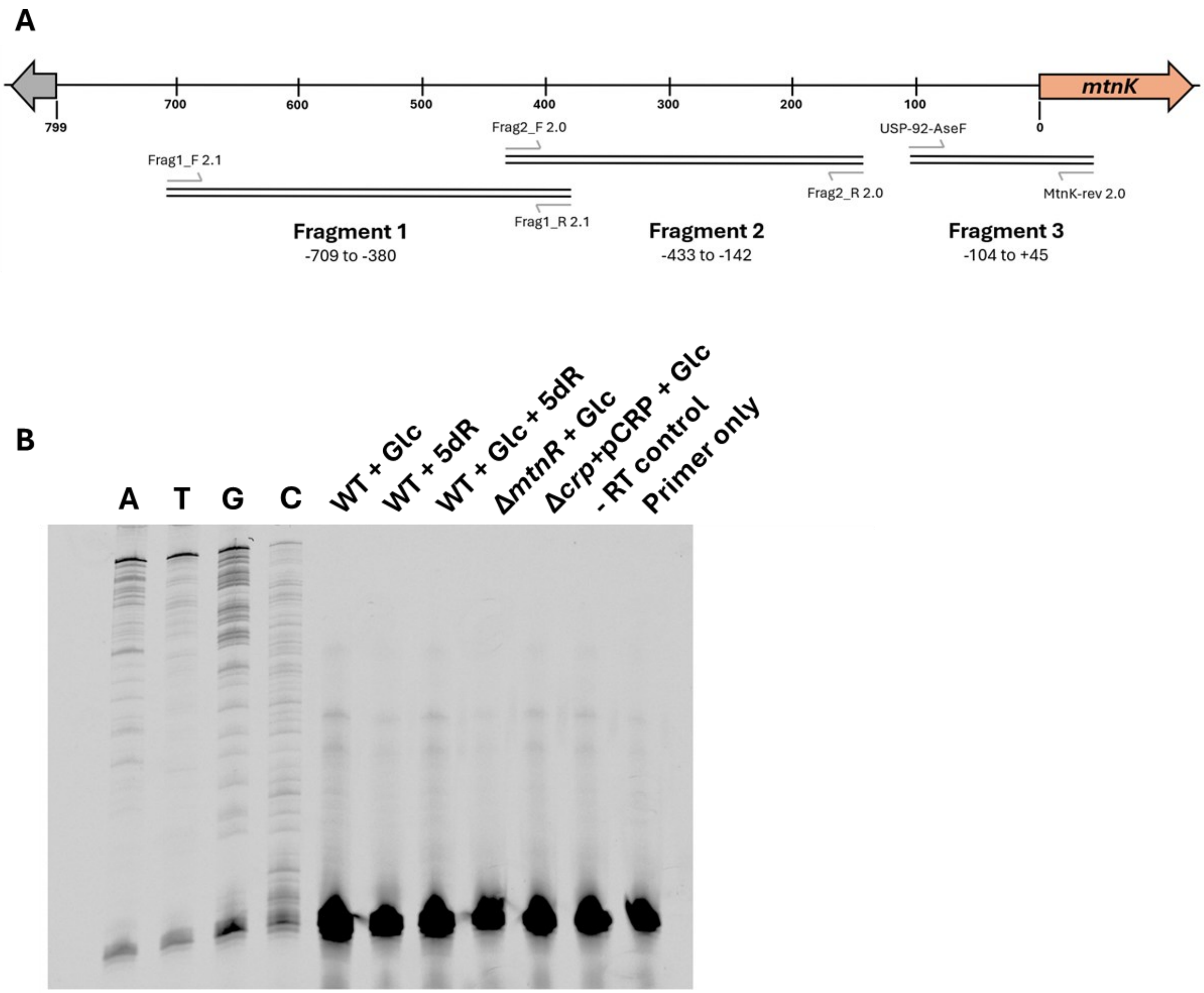

Suppl. Fig. S5. TSS mapping analysis. A) Visual representation of the upstream regions probed during the TSS mapping analysis. **B)** Primer extension analysis of the transcription start site for 381-709 bp upstream of *mtnK* (fragment 1). There are no identifiable transcription start sites associated with DHAP shunt operon expression in this region of the genome. Glc, glucose; 5dR, 5-deoxyribose; -RT control is primer extension of RNA from WT + Glc cells without any added reverse transcriptase; primer only is the primer extension reaction performed without any added RNA.

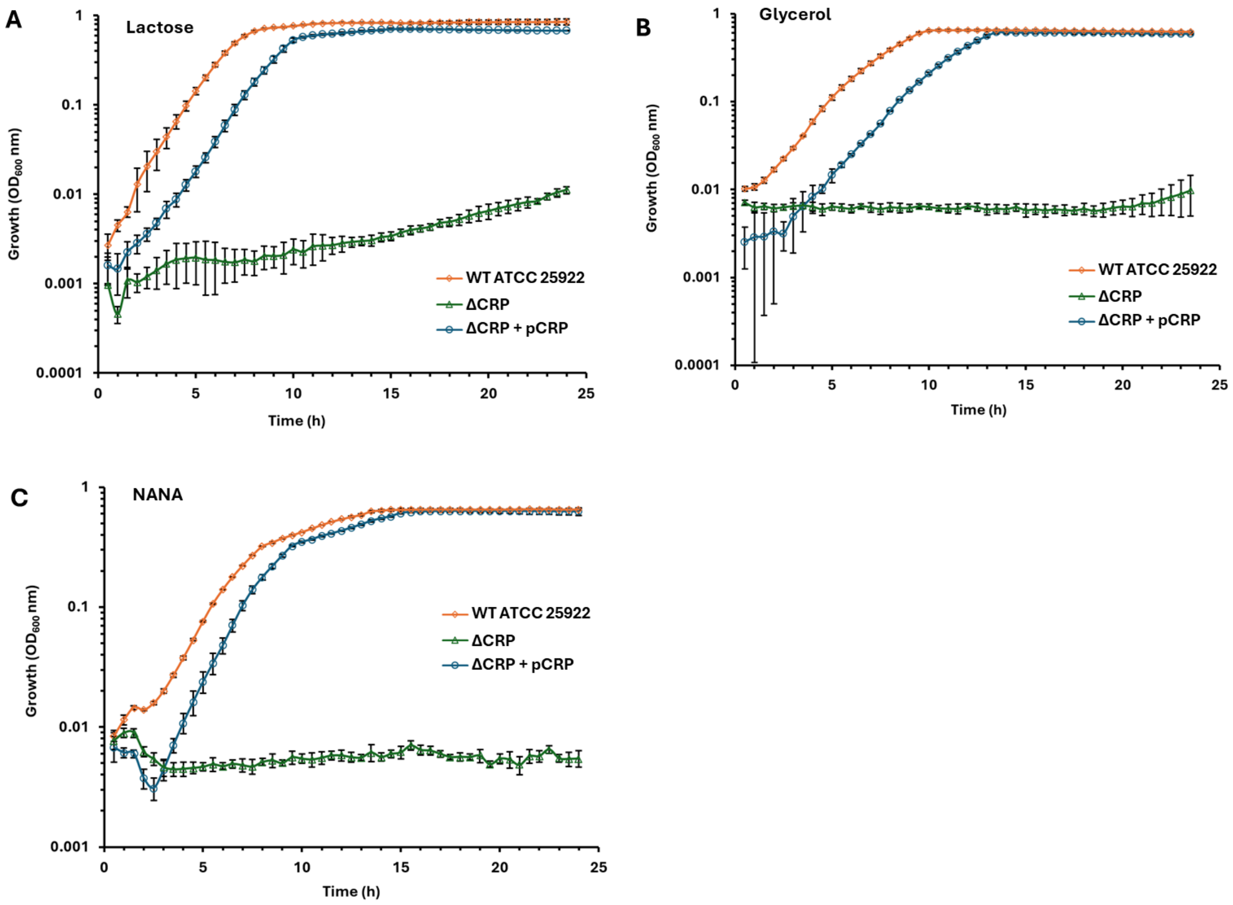

Suppl. Fig. S6. Growth of ATCC 25922 on various alternative growth substrates. Growth of wild type ATCC 25922 (diamonds), CRP deletion strain (Δ*crp*; triangles), and CRP deletion strain complemented with *crp* expressed from a plasmid (Δ*crp* + pCRP; circles) in in the presence of **A)** lactose, **B)** N-acetylneuraminic acid (NANA), and **C)** glycerol as the sole carbon source. Average and standard deviation error bars are for n = 5 independent biological replicates.

Supplementary Table S1: Plasmids used in this study and primers used for their construction

Plasmid	Description	Primer name	Sequence (5'-3'): restriction site underlined
pCRP	Expression vector for ATCC 25922 *crp* under regulation of tetracycline-inducible promoter	CRP-NdeI_F	ATAACCGCATATGGTGCTTGGCAAAC
		CRP-AgeI_R	ACTCTGACCGGTTTAACGAGTGCCGTAAAC
pET28-crp	Expression vector for hexahistidine-tagged ATCC 25922 *crp*	CRP-NdeI_F	ATAACCGCATATGGTGCTTGGCAAAC
		CRP-HindIII_R	ACTCTGAAGCTTTTAACGAGTGCCGTAAACGACGAT
placZ	Expression vector for ATCC 25922 *lacZ* under transcriptional control of IPTG-inducible promoter	lacZ-SpeI_F	GACTCACTAGTATGACTATGATTACGGATTCTCTGGCCG
		lacZ-AgeI_R	ATTGCACCGGTTTATTTTTGACACCAGACCAACTGGTAATGG
placZ799	Expression vector for ATCC 25922 *lacZ* under transcriptional control of 799 bp region upstream of DHAP shunt genes	UPS799-AseI_F	CGTACATTAATATTGAGCTACGATTTTCTTTCGTTGTCATC
		UPS0-SpeI_R	TGCGCTACTAGTTCTCCTTGTCTACACAGAATGATAATCAACTTG
placZ255	Expression vector for ATCC 25922 *lacZ* under transcriptional control of 255 bp region upstream of DHAP shunt genes	UPS255-AseI_F	AGCCGATTAATCTTCCTTTCGCCTAAGTTTGCCC
		UPS0-SpeI_R	TGCGCTACTAGTTCTCCTTGTCTACACAGAATGATAATCAACTTG
placZ169	Expression vector for ATCC 25922 *lacZ* under transcriptional control of 169 bp region upstream of DHAP shunt genes	UPS169-AseI_F	GCGATAATTAATGATGTTTTCCTCTGCTGATTCCTG
		UPS-BspEI_R	CACAGTTCCGGATTTTCAACGTTC
placZ126	Expression vector for ATCC 25922 *lacZ* under transcriptional control of 126 bp region upstream of DHAP shunt genes	UPS126-AseI_F	GGATCGATTAATCGACATCATTTTTAATATTATACAGTTCAAAAAATC
		UPS-BspEI_R	CACAGTTCCGGATTTTCAACGTTC
placZ66	Expression vector for ATCC 25922 *lacZ* under transcriptional control of 66 bp region upstream of DHAP shunt genes	UPS66-AseI_F	GCCTAGATTAATATTTGATAATGTATAGTTGTCACATGACAAG
		UPS-BspEI_R	CACAGTTCCGGATTTTCAACGTTC
placZUR1	Expression vector for ATCC 25922 *lacZ* under transcriptional control of 202 bp region far upstream of DHAP shunt genes	UPS799-AseI_F	CGTACATTAATATTGAGCTACGATTTTCTTTCGTTGTCATC
		UR1-SpeI_R	CGAACTACTAGTTCTCCTAATCACGAGGTCACAAAGGCGC
placZUR2	Expression vector for ATCC 25922 *lacZ* under transcriptional control of 365 bp region far upstream of DHAP shunt genes	UPS799-AseI_F	CGTACATTAATATTGAGCTACGATTTTCTTTCGTTGTCATC
		UR2-SpeI_R	CGAACTACTAGTTCTCCTCGATCGGCACTAACGATCAATATCG
placZUR3	Expression vector for ATCC 25922 *lacZ* under transcriptional control of 622 bp region far upstream of DHAP shunt genes	UPS799-AseI_F	CGTACATTAATATTGAGCTACGATTTTCTTTCGTTGTCATC
		UR3-SpeI_R	CGAACTACTAGTTCTCCTCCATGTAATTCATGTTGTTATGTCGCAAC
pMtnR	pTETTET expression plasmid for DHAP shunt transcription factor MtnR (*mtnR* gene product)	mtnR-NdeI_F	GATCTACATATGGATAGAGATCAAACAAACAG
		mtnR-SacI_R	GATCTAGAGCTCTTAGTGTGGTAATTCACACGCCAGCAAAAACTCTGCCG

Supplementary Table S2: PCR fragments and primers for their amplification

Fragment	Description	Primer name	Sequence (5'-3'): *E. coli* sequence underlined
qRT-lacZ	For synthesis of 254 bp fragment from *lacZ*	qRT_lacZ_F	GTTTGCCGTCTGAATTTGACCTGAGC
		qRT_lacZ_R	CAGCTCGCCGTACATCTGAACTTC
qRT-mtnK	Amplification of 286 bp fragment of ATCC 25922 *mtnK*	qRT_mtnK_F	CAGAAGCGCAGGCATTGTTACATGG
		qRT_mtnK_R	CCCTGTGTTTTGTTGTCCCAGAGC
qRT-rpoD	Amplification of 250 bp fragment of ATCC 25922 *rpoD*	qRT_rpoD_F	GGCCCATCTCTTGCAGCATCTG
		qRT_rpoD_R	AAATACACCAACCGTGGCTTGCAG
Primer Extension Fragment 1	Amplification of fragment 1 region as template for dideoxynucleotide ladder synthesis	Frag1_F 2.1	GCACTCGTCGCAGAATTCTTTATCGAGTGAGAGCC
		Frag1_R 2.1	CCCATCATCATGATGGAGCAACAGAGGTGAGTCTAACG
	For fluorescently labeled amplification of dideoxynucleotide sequencing ladders and primer extension for fragment 1	Frag1_R 2.1 CY5	Cy5-CCCATCATCATGATGGAGCAACAGAGGTGAGTCTAACG
Primer Extension Fragment 2	Amplification of fragment 2 region as template for dideoxynucleotide ladder synthesis	Frag2_F 2.0	TAAGAAGTGCGGAAACGTTAGACTCACC
		Frag2_R 2.0	TTACAGGAATCAGCAGAGGAAAACATCT
	For fluorescently labeled amplification of dideoxynucleotide sequencing ladders and primer extension for fragment 2	Frag2_R 2.0 CY5	Cy5-TTACAGGAATCAGCAGAGGAAAACATCT
Primer Extension Fragment 3	Amplification of fragment 3 region as template for dideoxynucleotide ladder synthesis	USP92-AseI_F	GCGATCATTAATTCTACATGTGTCACAAATAATTTTACATTTGATAATG
		MtnK-rev 2.0	GTCACATGTCAGCGGCTTGTAACCTGATGGAATTG
	For fluorescently labeled amplification of	mtnK-rev-Cy5	Cy5-GCTTGTAACCTGATGGAATTG

	dideoxynucleotide sequencing ladders and primer extension for fragment 3		
λRed-CRP	PCR fragment for markerless deletion of *crp* gene by λ-Red recombinase	Crp-F	ATAACCGCGCATGGTGCTTGGCAAACCGCAAACAGACCCGGTGTAGGCTGGAGCTGCTTC
		Crp-R	TCTGACGGGATTAACGAGTGCCGTAAACGACGATGGTTCATATGAATATCCTCCTTAGTTCCTATTCC
λRed-mtnR	PCR fragment for markerless deletion of *mtnR* gene by λ-Red recombinase	25922-MtnR_F	TGCAGCTATCATGGGGGTGTCCCGCGTCACGGTTGTCAAAGTGTAGGCTGGAGCTGCTTC
		25922-MtnR_R	GTTCATTCTTTTAGTGTGGTAATTCACACGCCAGCAAAAACATATGAATATCCTCCTTAGTTCCTATTCC
P1/2 fragment	Amplification of P1 and P2 promoters for EMSA of CRP binding	Footprint_F	CGCCTAAGTTTGCCCGTAATAATTCCCC
		Footprint_R	ATCCGTCATAACAGTTCTCCTTGTCTACACAG
		Footprint_R-Cy5	Cy5-ATCCGTCATAACAGTTCTCCTTGTCTACACAG
UR2 fragment	Amplification of region 579-799bp upstream of *mtnK*	UR2-799_F	ATTGAGCTACGATTTTCTTTCGTTGTCATCTTTGAG
		UR1-SpeI_R	CGAACTACTAGTTCTCCTAATCACGAGGTCACAAAGGCGC

Supplementary Table S3: DHAP shunt operon and 5'UTR sequences

>ATCC_25922_DHAP_shunt_5'UTR

ATTGAGCTACGATTTTCTTTCGTTGTCATCTTTGAGGCGTCTGATCGTGATAATCCTTCTTTCCCTGATTTCCTCATCGA

TATCGAATAAGCACTCGTCGCAGAATTCTTTATCGAGTGAGAGCCTCTGTTCAAGGACGCTTCTTTTTTTGTTGCAACGT

GCACATCTCTTCATCTTTCCGCGCCTTTGTGACCTCGTGATTTGGGGTACATGGAAAATGGGCGTAGCAAGTCTCAGCTA

GATAACTAACCGTAATAAAAGGGCTTTTCTGCGAGCAATGTAAGAGAAGGATAACCAGGCAGGTTGCAAGAAAGATAATC

GTTTTGATAGATCAGTTTATCGATATTGATCGTTAGTGCCGATCGATAAGAAGTGCGGAAACGTTAGACTCACCTCTGTT

GCTCCATCATGATGATGGGGCTGTTGGCCCTCGAATCTATCTGTCTATTAGCATCGGCATGACAAACACTATTTGTTTCC

ACAACAAAAGACAATAGAAAAGTACATATGTACAAAAAGTGTGATTTATTCTGTCACTTCAGTACTTCCTTTCGCCTAAG

TTTGCCCGTAATAATTCCCCGTGCAGTACTATTGTTGCGACATAACAACATGAATTACATGGTAATAATAGATGTTTTCC

TCTGCTGATTCCTGTAAAGATCATGACTCCATACGACATCATTTTTAATATTATACAGTTCAAAAAATCTACATGTGTCA

CAAATAATTTTACATTTGATAATGTATAGTTGTCACATGACAAGTTGATTATCATTCTGTGTAGACAAGGAGAACTGTT

>ATCC_25922_DHAP_Shunt_Operon

ATGACGGATTCAATTCCATCAGGTTACAAGCCGCTGACATGTGACACGCTGCCGGGTTATCTCTCGTCCAGACTGACCCCTTCATGCGAACCGGGA

GGGTTACCTGAAGAGTGGAAAGTTTCAGAAGTGGGGGACGGAAACCTGAACATGGTGTTTATCGTTGAGGGGACACATAAAACCATCATTGTAAAACA

AGCTCTGCCCTGGCTTCGTGCAGGGGGCGAAGGATGGCCTTTATCTCTGAGCCGTGCGGGCTTTGAGTACAACGTCTTATGTCAGGAAGCCAAGTA

CGCGGGTCACACACTGATTCCGCAGGTCTATTTTTACGACCCGGAAATGGCGCTGTTTGCCATGGAGTATCTGACTCCTCACGTGATTCTGCGTAAG

GAACTGATTAACGGTAAAAAATTCCCTAAACTAGCTGAAGATATCGGCAGATTTTTAGCACAGACTCTCTTCAATACGTCTGACATTGGCATGTCAGCAG

AACAGAAAAAAGCGCTTACTGCCGAGTTCGCGTTGAATCATGAGCTGTGCAAAATTACGGAAGATCTGATCTTCACAGAGCCCTATTACAACGCTGAA

CGGAATAACTGGACTTCTCCTGAGCTGGACGATGCCGTCCATAAGGCCTGGGCTGATGTAGAGATGATCCAGGTTGCCATGCGTTATAAGTACAAATT

TATGACAGAAGCGCAGGCATTGTTACATGGCGACCTTCATTCAGGCTCAATCATGGTGACCGACACGGATACCAAAGTGATTGATCCGGAGTTCGGTT

TCATGGGGCCAATGGCGTTTGATATCGGCAACTATATTGGCAACCTCCTGCTGGCGTACTTCTCACGCCCTGGGTGGGATGCGAATGAGCAACGTC

GCGCTGACTATCAGGAATGGCTGCTTCAGCAGATTGTCCAAACCTGGTCCGTTTTCACCCGGGAGTTCCGCCAGCTCTGGGACAACAAAACACAG

GGCGACGCGTGGCCGACAGAAATGTATCAACAGAACAGGGCCGCTCTTGAGGACGCACAGGATCAGTTTTTTGCCACGCTGCTTGAAGATTCCCT

GGTGAATGCCGGCATGGAAATGAATCGCCGGATCATTGGTTTTGCTGGCGTTGCCGAGCTGAAACAGATTGAAAATACAGAGCTTCGCGCAGGATGT

GAACGACGTGCATTGACCATGGCGCGCGATCTTATCGTCAATGCCCGCCAGTTTAAAAATATGGATTCCGTCATCCAGTCTGCGAAGGTTAAGTAAGA

GGTCATTATGAATATTAAAGGTAAACACTATCGTACGGTCTGGGTTTCCGGGGATGGAAAAGCGGTAGAAATCATCGATCAGACTAAGTTGCCTTTTAAG

TTCGAGGTGGTGGCGCTTACCTCTGCGGAAATGGCCGCTACGGCTATTCAGGAGATGTGGGTTCGTGGCGCTCCGCTTATTGGTGTCGTTGCAGCC

TATGGCATCGCGCTGGGAATGAATCACGATGCCAGTGACATGGGTCTGCAGCGTTACTATGACCTTCTTATCAAAACCCGACCAACGGCAATTAATCT

CAAATGGGCCCTCGACAGGATGATAGACACACTGAAGGATCTCTGTGTATCAGAGCGCAAGGATGTGGCCTGGGCGCTGGCGGCAGAAATTGCCG

AGGAAGATGTCGCGTTGTGTGAACAAATTGGATTACATGGTACAGAAGTCATCCGCGAAATAGCCCAGAAAAAACCGGCCGGGAGTGTGGTCAATAT

CCTGACACATTGTAACGCAGGCTGGCTGGCGACTGTAGACTGGGGAACCGCGCTTTCTCCCATCTACAAAGCGCATGAAAACGGAATCCCTGTTCA

CGTCTGGGTGGATGAAACGCGGCCACGTAATCAGGGCGGACTCACGGCATTTGAACTGGGCTCACACGGTATTCCTCACACCCTGATCGCCGACA
ACGCGGGTGGCCACCTGATGCAACACGGTGACGTTGATCTCTGCATTGTTGGGACCGATCGAACCACGGCCAGAGGGGATGTCTGTAATAAAATTG
GTACCTATCTTAAGGCGCTGGCGGCCCATGATAACCATGTTCCCTTCTACGTCGCGTTACCTTCTCCCACTATTGACTGGACTATCGAGGACGGAAAA
AGCATTCCGATTGAGCAACGTGACGGAAAAGAGCAGTCCCATGTCTATGGGATAAACCCTCAGGGAGAATTGAGCTGGGTCAATACCGCCCCTGAA
GGAACCCGTTGTGGGAACTACGCTTTCGACGTCACGCCTGCCCGATACATTACCGGCTTCATTACTGAGCGGGGAGTCTGTGCTGCCAGTAAGTCA
GCGCTGGCGGATATGTTTGCTGACCTGAAGAGTAAAGCACTCCAGGGGGAACAGCATTAAAACGGCGGCCATTGCTATTAAGGATCAGGAATGGAA
CGGATTAAGTTAGCAGAAAAAATCATTTCCACTTGTCGGGAAATGAATGCGTCAGGTCTTAATCAAGGAACATCAGGTAATGTGAGTGCCAGGTATACC
GGTGGCATGTTGATTACGCCGAGTGGGATCGCTTATTCAAAGATGACGCCGGACATGATTGTCTTTGTAGACGATAAGGGAATACCTGAAGCGGGTAA
AATACCATCAAGTGAATGGTTATTTCATCTGGCGTGTTATAAAGCCAGACCAGAATTGAATGCTGTCATACATACGCATGCGGTGAATTCCACGGCGGT
GGCAATACATAATCATTCAATTCCGGCCATTCATTATATGGTGGCGGTATCGGGGACGGATCATATTCCCTGTATTCCCTATTATACCTTTGGCAGTCCTG
AACTGGCCGACGGTGTATCTAAAGGAATCAGAGAAAGTAAGTCCTTGCTGATGCAGCACCACGGTATGCTGGCCATGGACGTCACGCTGGAAAAGA
CGTTGTGGCTGGCGGGTGAAACCGAGACGCTGGCTGATTTATACATCAAATGTGGCGGATTACATCACGATGTTCCCGTGCTGTCTGAAGCCGAAAT
GACCATCGTCCTTGAAAAATTCAAAACGTATGGTTTGAAAGCGTAATTACAGCTTTATTTTAATTAATAACATATGACAGCCATTCGCAGAATGGCTGCTAT
TCCCTCTGTCCACAACGATTGATTATAACTACCACAAGGAGTTATTTATGTTTATCGTGGAATCGTATGCTGTCGCCATAATAATGTGTTTTATTACCATGATT
TGCTGGGGTTCATGGGCGAATACAACTAAACTTGTCAGCAATAAAAAATGGGAGTTCCCTCTATTCTATTGGGATTATTCAATTGGATTATTGCTGTGTTCT
TTGTTATTTGCCTTCACGCTGGGTTCTATGGGCGAAGCCGGTCGCAGTTTTATACCTGATATTCAGCAGGCGAGCAGCAGTAGTCTGATGTCGGCAATA
CTGGCAGGGATTATCTTCAATATTTCCAATATTCTTCTGGTCGCTTCTATTAATCTTGCCGGTATGGCGGTAGCTTTTCCTGTGGGTGTGGGGCTGGCAC
TGGCTCTGGGTGTGATCACCACCTATATCGGCAATCCGCAGGGCGATCCGCTCATTCTGTTCCTCGGTGTCGCCTGCGTTGTGAGCGCCATTATTTTT
ACCGCGATAGCCTATGGTCGTGTCACGCAGGAGGCGGATAAATCTCGCCGTAATAAAGGTCTCATTACTGCCATTCTGGCGGGTATCATCATGGGAT
GGTTTTTCCGCTTCTTGGCGGACTCCATGTCTGACAATTTCAGCCAGCCTGCCAGCGGTCTGATGACGCCTTACTCAGCACTTGTTCTGTTTGCCGTG
GGGCTGTTCTTGTCGAACTTTGTCCTTAACACGCTGGTCATGAAAAAACCTATTTCAGGAGAACCGGTCAACGGGAAAATGTATTTTTCCGGGAGCTTA
CGGGATCATGTTTGCGGCTGGCTGGGCGGTATGATTTGGTGTGTCGGTCTGGCATTCAGTCTGATTGCGTCTGGTCAGGCGGGGTATGCCATTTCCTA

CGGTCTGGGACAGGGGGCCACAATGATTGCCGTGATCTGGGGAGTCTTCATCTGGAGAGAATTTGCCAGTGCGCCAGCCGGAACCAATAAACTGC
TGCTGACCATGTTTATTTCATACATTGTGGGTATTGTTCTTATTATCGCCGCTAATCAATAA

Supplementary Dataset Captions:

Supplementary dataset S1: Differential expression results of transcriptomic analyses – This excel workbook contains results from transcriptomic analyses comparing different carbon growth conditions. **Sheet1-WT 25922 5dR vs glucose:** Differentially expressed genes between 5dR and glucose grown cultures of WT E. coli ATCC 25922. Includes genomic gene location, gene name, base mean expression, log2 fold-change, lfcSE, and adjusted p-values. **Sheet2-mtnR vs WT 25922 glucose:** Differentially expressed genes between ΔmtnR and WT ATCC 25922 grown on glucose. Includes genomic gene location, gene name, base mean expression, log2 fold-change, lfcSE, and adjusted p-values. **Sheet3- WT 25922 glc- vs glc+:** Differentially expressed genes between carbon starvation conditions and replete glucose cultures of WT ATCC 25922. Includes genomic gene location, gene name, base mean expression, log2 fold-change, lfcSE, and adjusted p-values. **Sheet4-Gene categories:** The categorization of genes in the ATCC 25922 genome based on annotations by UniProt and NCBI. Used in Figure 2D and Supplemental Figure S2

Supplementary dataset S2: Computational analysis results of promoter region alignment – This excel workbook contains results of each computational analysis identifying coverage and identity of aligned promoter regions. **Sheet1-%coverage groups:** Data used in Supplemental Figure S1A. Sheet provides sample IDs for each E. coli genome or contig associated with a percent coverage compared to the ATCC 25922 5'UTR reference sequence (see Supplementary Table S3). Percent coverage and visualization preformed using Python (see Supplementary Methods - Python code used for percent coverage calculation and visualization). **Sheet2-Upstream DHAP shunt alignment:** Data used in Figure 1B and Supplemental Figure S1A. Sheet provides numerical values for subject's percent identity to the 5'UTR reference in addition to the percent identity to ATCC 25922 DHAP shunt operon (see Supplementary Table S3). Percent coverage and visualization preformed using Python (see Supplementary Methods - Python code used for sequence alignment and visualization). **Sheet3-Upstream alignment:** Data used for Figure 1B, filtering out samples from Sheet2 that had less than 70% of a complete DHAP shunt gene cluster (putative permease not included in total percentage).

Supplementary dataset S3: Sequences for promoter region alignment – Sequences identified by BLAST using the 5'UTR from ATCC 25922 queried against E. coli (taxid562). To increase the number of dissimilar sequences identified, discontinuous megablast was selected with the algorithm parameters adjusted to increase the number of target sequences and reduce the penalties for mismatched bases and gaps in sequences. The curated sequences were used for all computation analyses involving the promoter region alignment.

Supplementary References:

1. Schneider K, Asao M, Carter MS, Alber BE. 2012. Rhodobacter sphaeroides uses a reductive route via propionyl coenzyme A to assimilate 3-hydroxypropionate. *J Bacteriol* 194:225-32.
2. Dangel AW, Tabita FR. 2015. Amino acid substitutions in the transcriptional regulator CbbR lead to constitutively active CbbR proteins that elevate expression of the cbb CO2 fixation operons in Ralstonia eutropha (Cupriavidus necator) and identify regions of CbbR necessary for gene activation. *Microbiology (Reading)* 161:1816-1829.

3. Dubbs JM, Tabita FR. 1998. Two functionally distinct regions upstream of the cbbI operon of Rhodobacter sphaeroides regulate gene expression. *J Bacteriol* 180:4903-11.

4. Feng Y, Cronan JE, 2012. Crosstalk of Escherichia coli FadR with Global Regulators in Expression of Fatty Acid Transport Genes. *PLOS One* 7:e46275

5. Gilbert J, Valldeperas M, Dhayal SK, Barauskas J, Dicko C, Nylander T. 2019. Immobilisation of β-galactosidase within a lipid sponge phase: structure, stability and kinetics characterisation. *Nanoscale* 11:21291-21301.

6. Milne I, Stephen G, Bayer M, Cock PJ, Pritchard L, Cardle L, Shaw PD, Marshall D. 2013. Using Tablet for visual exploration of second-generation sequencing data. *Brief Bioinform* 142:193-202.
